# Tissue distribution and integrated pharmacokinetic properties of major effective constituents of oral *Gegen*-*Qinlian* decoction in mice

**DOI:** 10.3389/fphar.2022.996143

**Published:** 2022-10-11

**Authors:** Jing-Ze Lu, Dan-Dan Hong, Dan Ye, Sheng Mu, Rong Shi, Yu Song, Chu Feng, Bing-Liang Ma

**Affiliations:** ^1^ Department of Pharmacology, Shanghai University of Traditional Chinese Medicine, Shanghai, China; ^2^ Experiment Center for Science and Technology, Shanghai University of Traditional Chinese Medicine, Shanghai, China; ^3^ Department of Dermatology, Longhua Hospital Shanghai University of Traditional Chinese Medicine, Shanghai, China; ^4^ Department of Surgery, Putuo District People’s Hospital, Shanghai, China

**Keywords:** gegen-qinlian decoction, effective constituents, integrated pharmacokinetics, tissue distribution, LC-MS/MS

## Abstract

*Gegen-Qinlian* decoction (GQD) is a classic traditional Chinese medicine (TCM) formula. GQD is effective against colon or liver-related diseases including ulcerative colitis, non-alcoholic fatty liver, and type 2 diabetes. In this study, a liquid chromatography-tandem mass spectrometry method was developed, validated, and then applied to reveal the tissue distribution and integrated pharmacokinetic properties of major effective constituents of oral GQD in mice. The established method was quick, sensitive, and accurate enough to analyze GQD constituents in plasma and tissue homogenate samples quantitatively. According to their concentrations in the portal vein, systemic circulation, liver and colon samples of the mice after oral administration of GQD, the concentration-time curves of the constituents were respectively plotted. The results showed that daidzein, baicalin, and baicalein had relatively high exposure levels in the livers, while puerarin, berberine, epiberberine, coptisine, palmatine, jatrorrhizine, magnoflorine, glycyrrhizic acid, and glycyrrhetinic acid were enriched in the colons. Given that these constituents have significant biological activity, they could be regarded as the major effective constituents of GQD in treating colon or liver-related diseases, respectively. In addition, the integrated pharmacokinetic properties of GQD were studied. The GQD “integrated constituent” reached peak concentration at 4.0 h in the portal vein, the systemic circulation, the livers, and the colons, with half-lives of 1.5–4.1 h and mean retention time of 4.5–6.3 h, respectively. Furthermore, the concentration of the GQD “integrated constituent” in the colons was approximately 10 times higher than that in the livers, both of which were much higher than that in the systemic circulation, indicating its accumulation in these tissues, especially in the colons. In conclusion, the tissue distribution and integrated pharmacokinetic properties of oral GQD were revealed in the study. The results of the tissue distribution study would contribute to identifying the major target tissues and effective constituents of GQD, while the results of the integrated pharmacokinetic study would help to explain the pharmacokinetic properties of oral GQD as a whole.

## 1 Introduction

*Gegen-Qinlian* decoction (GQD) is a classic traditional Chinese medicine (TCM) formula, which is composed of four TCMs including *Pueraria Lobatae Radix* (*PR*), *Scutellaria Radix* (*SR*), *Coptidis Rhizoma* (*CR*), and *Glycyrrhizae Radix* et *Rhizoma Praeparata Cum Melle* (*GR*) according to the ratio of 8: 3: 3: 2 (Hai and Wei, 2009). Owing to its significant anti-pathogenic microorganisms (bacteria and viruses), anti-inflammatory, and antioxidant bioactivities, GQD is effective against colon or liver-related diseases including diarrhea (Liu et al., 2019), ulcerative colitis (Zhao et al., 2020), non-alcoholic fatty liver (Zhang et al., 2020), and type 2 diabetes (Cao et al., 2020). GQD is one of the few TCM formulas that have been proved curative through strict clinical trials. In a randomized, double-blind, placebo-controlled clinical trial, treatment with oral GQD for 12 weeks dose-dependently improved symptoms and significantly reduced blood glucose and glycosylated hemoglobin levels in patients with type 2 diabetes (Xu J. et al., 2015).

The effective material basis, a key issue for understanding the pharmaceutical nature of GQD, remained to be identified (Lu et al., 2021). The effective material basis of a TCM should not only have significant bioactivity but also have relatively high *in vivo* exposure levels. After oral administration, 107 prototype constituents and 67 metabolites of GQD were qualitatively detected in the plasma, urine, bile, and feces of rats (Liu et al., 2018). In addition, the pharmacokinetics of GQD in the systemic circulation of the rats received oral GQD were reported (Qiao et al., 2018). However, the pharmacokinetic properties of GQD constituents in their sites of action, i.e., target tissues, were unclear.

Studies have shown that some GQD constituents have significantly different pharmacokinetic properties between in the systemic circulation and target tissues. For example, the oral bioavailability of berberine was as low as 0.36% in rats (Liu et al., 2010), suggesting that its local concentration and bioactivity in the intestinal tract may have pharmacological value. Furthermore, after absorption, its concentration in the liver was much higher than that in the systemic circulation (Li et al., 2018), which explained its hepatoprotective effects (Bansod et al., 2021). According to pharmacological studies, GQD was effective on regulating pathogenic microorganisms such as bacteria and viruses in the intestinal tract (Liu et al., 2019), relieving intestinal inflammation (Zhao et al., 2020), repairing tight junctions between intestinal epithelial cells (Zhao et al., 2020), and regulating liver glucose (Cao et al., 2020) and lipid (Zhang et al., 2020) metabolism. Therefore, the distribution and dynamic change of the effective GQD constituents in the potential target tissues including the liver, the intestinal tract, especially the colon, would be closely related to the pharmacological effects of GQD. Conversely, pharmacokinetic studies focusing on the target tissues would help to reveal the major effective constituents of GQD.

Furthermore, due to the significant difference between GQD constituents, it was challenging to interpret and utilize their pharmacokinetic parameters comprehensively. For example, elimination half-life (T_1/2_) is one of the basic pharmacokinetic parameters to establish a dosage regimen of drugs. However, it was not convictive enough to formulating the dosing regimen of GQD based on the T_1/2_ value of any single constituent or single type of constituents. A strategy of “integrated pharmacokinetic study of multiple constituents of TCM” was put forward to resolve the common problem faced by TCMs (Li et al., 2008; Hao et al., 2009; Shi P. et al., 2018). Above all, the pharmacokinetic weight coefficients of the constituents are respectively calculated based on their values of area under the concentration-time curve (AUC), which reflect their exposure levels *in vivo*. Then, the concentration at each time point of the “integrated constituent” is calculated by summing up the “integrated concentration” (real concentration times pharmacokinetic weight coefficient) of each constituent. Finally, the pharmacokinetic parameters of the “integrated constituent” are calculated according to its concentration-time (C-T) curve. The integrated pharmacokinetics of classical TCM formulas including *Huanglian*-*Jiedu* decoction (Zhu et al., 2012) and *Jiao-Tai* pill (He et al., 2014) were reported. Studies on the integrated pharmacokinetics of GQD would help to explain its pharmacokinetic properties as a whole.

In this study, in order to simultaneously determine the concentrations of 19 major GQD constituents in biological samples (plasma, and the homogenate of the livers and the colons), a rapid and sensitive quantitative analysis method based on liquid chromatography-tandem mass spectrometry (LC-MS/MS) was developed. The method was fully validated with plasma matrix and partially validated with liver homogenate matrix. The integrated pharmacokinetics of the GQD constituents in blood (the portal vein, the systemic circulation) and major target tissues (the colon, the liver) were then comparatively studied in mice. Based on the obtained data, the tissue distribution and the integrated pharmacokinetic properties of GQD were revealed.

## 2 Materials and methods

### 2.1 Materials

The crude drug of *PR* (batch number 190910), *SR* (batch number 191207), *CR* (batch number 190820), and *GR* (batch number 191118) were all purchased from Shanghai Kangqiao Herbal Pieces Co., Ltd. (Shanghai, China). According to the Pharmacopeia of the People’s Republic of China (2020 edition), the crude drug were identified as the dried root of *Pueraria lobata* (Willd.) Ohwi, *Scutellaria baicalensis* Georgi, *Coptis chinensis* Franch., and *Glycyrrhiza uralensis* Fisch. respectively. The voucher specimens were deposited in Shanghai University of Traditional Chinese Medicine.

The reference compounds of berberine hydrochloride, baicalein, baicalin, wogonin, daidzein, puerarin, glycyrrhizic acid, glycyrrhetinic acid, liquiritin, isoliquiritin, and jatrorrhizine hydrochloride were purchased from Shanghai Yuanye Biological Co., Ltd. (Shanghai, China). The reference compounds wogonoside, daidzin, palmatine hydrochloride, coptisine hydrochloride, mycophenolic acid, naringin, and berberrubine were purchased from Dalian Meilun Biotechnology Co., Ltd (Dalian, China). The reference compound liquiritigenin was purchased from Chengdu Must Bio-technology Co., Ltd (Chengdu, China). The reference compounds magnoflorine, demethyleneberberine, and epiberberine were purchased from National Institutes for Food and Drug Control (Beijing, China). The purity of all the reference compounds was greater than 98%.

Methanol and acetonitrile were the products of Burdick and Jackson (Ulsan, Korea). Ammonium formate, formic acid, and acetic acid were purchased from Thermo Fisher Scientific Inc. (Massachusetts, USA). All the materials were HPLC grade.

### 2.2 Animals

Clean grade male and female Kunming mice (weighted at 22–24 g) were used in this study. The mice were purchased from Beijing Vital River Laboratory Animal Technology Co., Ltd. (Beijing, China), which has a production license number of SCXK (Beijing) 2016-0011. The mice were raised in the experimental animal center of Shanghai University of traditional Chinese medicine, which has the experimental license of SYXK (Shanghai) 2020-0009. The ambient temperature of the feeding room was maintained at 22–24°C, with a 12-h dark/light cycle. Before the experiment, all animals were fasted for 12 h but could drink freely. The animal experiments were approved (PZSHUTCM211129015) by and conducted in accordance with the guideline of the Institutional Animal Care and Use Committee of Shanghai University of Traditional Chinese Medicine.

### 2.3 Validating of a LC-MS/MS method

#### 2.3.1 Preparation of solutions of reference compounds

Stock solutions of the reference compounds were prepared in dimethylsulfoxide at the concentration of 1.0 mg/mL. A series of standard working solutions of the reference compounds, including those of quality control (QC) samples, were prepared by diluting the stock solutions with methanol. The internal standards (ISs), naringin and mycophenolic acid, were dissolved in methanol (1.0 mg/ml) and then diluted with methanol to obtain the working solutions that contained 20 μg/ml naringin and 150 ng/ml mycophenolic acid, respectively. All the solutions were stored at 4°C and were brought to room temperature (about 22°C) before use.

#### 2.3.2 Pretreatment of plasma and tissue homogenate samples

Blood of the mice (blank or received oral GQD) was mixed fully with heparin and 5% volume of ascorbic acid solution (4 mg/ml, for increasing the stability of the constituents). Then the mixed solution was centrifuged at 4°C for 10 min at the speed of 8,000 rpm to obtain plasma. The plasma samples for calibration curves and QCs were prepared by adding 2 µl the corresponding mixed working solutions of the reference constituents to 38 µl blank plasma.

Sequentially, 10 µl mixed working solution of the ISs, 20 µl ammonium formate aqueous solution (100 mM), and 120 µl acetonitrile were added to 40 µl the plasma samples (for standard curves or QCs, or the plasma samples obtained from mice that received oral GQD). After shaking for 3 min, the mixtures were centrifuged for 10 min at 4°C at the speed of 14,000 rpm. 180 µl supernatant was collected and then dried with nitrogen flow at 40°C. The residue was dissolved in 90 µl solution composed of methanol, water, and acetic acid at the volume ratio of 70: 29.9: 0.1. The solution was mixed by shaking for 1 min, and then centrifuged for 10 min at 4°C at the speed of 14,000 rpm. The supernatant was collected and injected in the LC-MS/MS system for analysis.

The liver and the colon homogenates were prepared with 10 times (V: W) the volume of ascorbic acid solution (200 μg/ml). Then the homogenates were pretreated as plasma to obtain the tissue samples for LC-MS/MS analysis.

#### 2.3.3 Instrumentation and conditions

The LC-MS/MS analysis was performed using an ACQUITY UPLC (Waters, USA) equipped with an autosampler, and an API QTRAP 6500 + mass spectrometer (Applied Biosystems, USA) equipped with an electrospray ionization source.

The chromatographic separation was performed using an ACQUITY BEH C_18_ column (2.1 × 100 mm, 1.7 μm), a product of Waters technology (Shanghai) Co., LTD (Shanghai, China). The mobile phase was composed of phase A (5 mM ammonium acetate, containing 0.1% formic acid) and phase B (methanol). The elution gradient program was set as below: 0 min, 10% B; 1 min, 40% B; 5 min, 60% B; 10 min, 70% B; 12 min, 90% B; 18 min, 90% B; 18.1 min, 10% B; 20 min, 10% B. The flow rate was 0.3 ml/min. The column temperature and autosampler temperature were maintained at 40 and 4°C, respectively. The injection volume was 2 μl.

The working parameters for the mass spectrometer were set as follows: ion-source temperature 500°C; spray voltage 4500 V (+) or 4500 V (-), respectively; atomization gas pressure 50 psi; auxiliary gas pressure 50 psi, and curtain gas pressure 35 psi. Naringin and mycophenolic acid were used as the positive and negative IS, respectively. Quantification was performed using the multiple reactions monitoring mode. The parameters including declustering potential, collision energy, collision cell exit potential, and transitions for the analytes were listed in Supplementary Table S1.

#### 2.3.4 Linear ranges of the constituents and methodological validation

The calibration plasma samples were prepared by adding the mixed working solutions of the constituents to blank plasma at eight concentrations. The linear ranges of the constituents were listed in Table 1. In the liver homogenate samples, the linear range of baicalein was within 5.0–640.0 ng/ml, glycyrrhizic acid was within 25.0–3,200.0 ng/ml, and the linear ranges of other constituents were the same as those in the plasma samples.

**TABLE 1:** Standard curves, linear ranges, and lower limit of quantification (LLOQ) of the constituents of *Gegen-Qinlian* decoction in blank mouse plasma (n = 6).

Constituents	Regression equations	r	Linearity ranges (ng/ml)	LLOQs (ng/ml)
Puerarin	y = 0.00867x+0.0554	0.9952	2.5–320.0	2.5
Daidzein	y = 0.012x+0.0719	0.9961	2.0–256.0	2.0
Daidzin	y = 0.0122x+0.02	0.9969	1.6–200.0	1.6
Baicalin	y = 0.0139x-0.0181	0.9964	5.0–640.0	5.0
Wogonoside	y = 0.00366x+0.0156	0.9963	5.0–640.0	5.0
Baicalein	y = 0.00617x-0.00792	0.9952	3.9–500.0	3.9
Wogonin	y = 0.0512x+0.033	0.9963	1.0–128.0	1.0
Berberine	y = 0.0647x+1.42	0.9957	1.0–128.0	1.0
Palmatine	y = 0.323x+1.69	0.9971	0.3–32.0	0.3
Coptisine	y = 0.0309x+0.106	0.9968	0.5–64.0	0.5
Epiberberine	y = 0.0217x+0.399	0.9954	1.0–128.0	1.0
Magnoflorine	y = 0.22x+0.0403	0.9953	0.5–64.0	0.5
Demethyleneberberine	y = 0.0403x+0.00894	0.9952	1.0–128.0	1.0
Berberrubine	y = 0.11x-0.0508	0.9958	0.8–100.0	0.8
Jatrorrhizine	y = 0.0665x+0.203	0.9975	0.5–64.0	0.5
Liquiritin	y = 0.111x+0.11	0.9960	3.1–400.0	3.1
Liquiritigenin	y = 0.0812x+1.16	0.9974	3.1–400.0	3.1
Glycyrrhizic acid	y = 0.00101x-0.00818	0.9951	30.0–3,840.0	30.0
Glycyrrhetinic acid	y = 0.0027x+0.0138	0.9956	25.0–3,200.0	25.0

Full and partial methodological validation were performed respectively for the plasma and the liver homogenate samples in terms of specificity, calibration curves, lower limit of quantification (LLOQ), precision and accuracy, carry over, recovery, matrix effect, dilution linearity, and stability (see Methodological validation in supplementary materials).

### 2.4 Preparation and quality control of GQD extract

The herbal pieces were extracted with 10 times volume of boiling water for twice. For the first time, the herbal piece of *PR* was decocted separately for 20 min, and then decocted together with *SR*, *CR*, and *GR* at the weight ratio of 8: 3: 3: 2 for another 1 h. The aqueous extract was filtered through four layers of gauze. Then the residue was extracted again with 10 times volume of water for 1 h. The filtrates obtained from the two decoctions were combined and vacuum-dried at 60°C to obtain the powder of GQD extract.

The contents of 17 constituents in the GQD extract were measured. Briefly, the extract powder was dissolved in the water solution of methanol (50%, V/V) at the concentration of 1 mg/ml. The solution was treated with ultrasound for 60 min and then centrifuged at 14, 000 rpm for 10 min. The obtained supernatant was diluted and mixed with the solution of the ISs, and then injected into a LC-MS/MS system for quantitative analysis. The instrumentation and conditions was mostly the same as those described in section “2.3.3”. However, the elution gradient program was set as below: 0 min, 10% B; 1 min, 10% B; 5 min, 40% B; 11 min, 60% B; 15 min, 70% B; 24 min, 90% B; 26.1 min, 90% B; 28 min 10% B. The mass spectrometry parameters of the constituents and ISs were listed in Supplementary Table S2. The LC-MS/MS method was established by our group and had been verified to meet the requirements of quantitative analysis of the constituents in GQD extract.

### 2.5 Pharmacokinetics of the major constituents of oral GQD in mice

Fifty-four Kunming mice were randomly divided into nine groups according to body weight and gender (3 male and three female in each group). Each group was orally administered with water solution (0.2 ml/10 g body weight) of the GQD extract powder at the dosage of 6.1 g/kg body weight. The dosage of GQD extract powder equals to 18 g/kg herbal pieces of GQD, which is equivalent to its clinical dosage. At each designed time point (0.08, 0.25, 0.5, 1, 2, 4, 8, 12, and 24 h) after oral administration, six mice were anesthetized with ether, and blood was collected from the portal vein (about 0.2 ml, aspirated in a 1 ml heparinized syringe) and then the systemic circulation (about 0.2 ml, collected in heparinized tubes after removal of the eyeball) of the mice to prepare plasma samples, and the livers and the colons were removed to prepare respective homogenate. After pretreatment, the concentrations of 19 major constituents of GQD in each sample were measured by the validated LC-MS/MS method (see “2.3”). Then the C-T curves of the constituents in the plasma and the tissue homogenates were respectively drawn and their pharmacokinetic parameters were respectively calculated.

### 2.6 Integrated pharmacokinetics of the constituents

Above all, the AUC_0–24 h_ values of the constituents were summed up to obtained the ∑AUC_0–24 h_ value (Eq. 1). Then, the ratio of the AUC_0–24 h_ value of each constituent to ∑AUC_0–24 h_ was calculated and defined as the weight coefficient of the constituent (W_j_, Eq. 2). After that, the plasma concentrations of the constituents at each time point were multiplied by respective weight coefficients and summed up to obtain the concentration of the “integrated constituent” at each time point (C_t_, Eq. 3). Finally, the pharmacokinetic parameters of the “integrated constituent” were calculated based on its C-T curves.

$$\begin{array}{l} \sum {AUC}_{0-24\;h}={AUC}_{0-24\;h},\;\text{puerarin}+{AUC}_{0-24\;h},\;\text{daidzein}{+\text{AUC}}_{0-24\;h},\;\text{daidzin}\;{+\text{AUC}}_{0-24\;h,\;}\text{baicalin}\;{+\text{AUC}}_{0-24\;h},\;\text{wogonoside}{+\text{AUC}}_{0-24\;h},\;\text{baicalein}{+\text{AUC}}_{0-24\;h},\;\\ {AUC}_{0-24\;h},\text{wogonin}\;{+\text{AUC}}_{0-24\;h},\;\text{berberine}{+\text{AUC}}_{0-24\;h},\;\text{palmatine}{+\text{AUC}}_{0-24\;h},\;\text{coptisine}\;{+\text{AUC}}_{0-24\;h},\;\text{epiberberine}{+\text{AUC}}_{0-24\;h},\;\text{magnoflorine}{+\text{AUC}}_{0-24\;h},\;\text{demethyleneberberine}{+\text{AUC}}_{0-24\;h},\;\text{berberrubine}\;{+\text{AUC}}_{0-24\;h},\;\text{jatrorrhizine}+{\text{AUC}}_{0-24\;h},\;\text{liquiritin}\;+{\text{AUC}}_{0-24\;h},\;\text{liquiritigenin}\;{+\text{AUC}}_{0-24\;h},\;\text{glycyrrhizic}\;\text{acid}\;{+\text{AUC}}_{0-24\;h},\;\text{glycyrrhetinic}\;\text{acid} \end{array}$$ (1)

$$W_{j}{=\text{AUC}}_{j\;0-24\;h}/\sum {\text{AUC}}_{0-24\;h}$$ (2)

$$C_{t}=\sum \left(W_{j}\times C_{j}\right)$$ (3)

In Eq. 2, j represented one of the 19 GQD constituents; W_j_ represented the ratio of the AUC_0–24 h_ value of a GQD constituent to ∑AUC_0–24 h_. In Eq. 3, C_j_ represented the real concentration of a GQD constituent at a designated time point, while C_t_ represented the concentration of the “integrated constituent” of GQD at the designated time point.

### 2.7 Data analysis

A non-compartmental analysis using the WinNonlin^®^ software (Pharsight, CA, USA) was performed to obtain the pharmacokinetic parameters including peak concentration (T_max_), time to reach peak concentration (C_max_), area under the concentration time curve (AUC_0–24 h_), elimination half-life (T_1/2_), and mean retention time (MRT). It should be noted that the pharmacokinetic parameters of a GQD constituent was calculated based on its average concentration in six mice at each time point. The results were expressed as mean ± SD.

## 3 Results

### 3.1 Methodological validation for plasma samples

There was no endogenous interference of the GQD constituents and the ISs in the blank plasma matrix (Supplementary Figure S1). The LLOQ (Table 1) was 1.6–2.5 ng/ml for three constituents (puerarin, daidzein, and daidzin) of *PR*, 1.0–5.0 ng/ml for four constituents (baicalin, baicalein, wogonoside, and wogonin) of *SR*, 0.3–1.0 ng/ml for eight constituents (berberine, epiberberine, palmatine, coptisine, jatrorrhizine, magnoflorine, demethyleneberberine, and berberrubine) of *CR*, 3.1 ng/ml for two flavonoids (liquiritin and liquiritigenin) and 25.0–30.0 ng/ml for two saponins (glycyrrhizic acid and glycyrrhetinic acid) of *GR*. As shown in Table 1, the ratio of the upper limit of quantification (ULOQ) to the LLOQ of each constituent was 128 times, and the linearity of each constituent was good within its concentration range. As shown in Supplementary Table S3, at the concentration of LLOQ, the intraday precision of each constituent was 4.2%–19.1%, the interday precision was 1.4%–14.8%, the intraday accuracy was 84.3%–118.6%, while the interday accuracy was 91.2%–114.4%; as for other concentrations, the intraday precision was 1.0%–12.5%, the interday precision was 0.8%–12.0%, the intraday accuracy was 85.5%–114.3%, while the interday accuracy was 90.1%–109.8%. The average peak area of each constituent in the double blank plasma samples was less than 12.1% of its corresponding peak area in the LLOQ samples, while the response of the ISs was less than 2.7% of that in the LLOQ samples, indicating there were no significant carry over for both the constituents and the ISs (Supplementary Table S4). As shown in Supplementary Table S5, the average recovery of each constituent was 85.1%–112.1% with RSD lower than 13.7%; the matrix effect was 86.5%–114.6% with RSD lower than 11.4%, indicating acceptable recovery and matrix effect of the constituents. As shown in Supplementary Table S6, after being placed at -80°C for 7 days, the accuracy of each constituent was 86.0%–114.2% with RSD lower than 13.4%; after being placed at 4°C for 24 h, the accuracy was 88.0%–113.5% with RSD lower than 14.5%; after being placed at room temperature (about 22°C) for 2 h, the accuracy was 85.7%–113.0% with RSD lower than 13.1%, indicating that the constituents were stable during the sampling and analyzing procedure. In addition, the accuracy of the concentrations measured after 10 times dilution was 102.2%–114.6% with RSD lower than 11.7% (Supplementary Table S7), indicating good dilution linearity of the constituents.

### 3.2 Partial methodological validation for liver samples

The LLOQs (Supplementary Table S8) were mostly the same as those in plasma samples, except that the LLOQ of glycyrrhizic acid and baicalein was 25.0 and 5.0 ng/ml, respectively. In addition, there was no endogenous interference of the GQD constituents and the ISs in the blank liver homogenate matrix (Supplementary Figure S2); the linearity of each constituent was good within its concentration range (Supplementary Table S8); the intraday precision and accuracy of each constituent was acceptable (Supplementary Table S9); there were no significant carry over for both the constituents and ISs (Supplementary Table S10); the constituents had good recovery and negligible matrix effect (Supplementary Table S11); the constituents were stable during the sampling and analyzing procedure (Supplementary Table S12).

### 3.3 Preparation and contents of the major constituents in GQD extract powder

A total of 992 g herbal pieces were decocted with a yield of 33.9% to obtain 336 g of GQD extract powder. The total and extracted ion chromatograms of GQD constituents in water solution were shown in Supplementary Figure S3. As shown in Table 2, the contents of 17 major constituents were measured in GQD extract powder. Baicalin had the highest content among the constituents. As for the three constituents of *PR*, puerarin had the highest content, and the content of daidzin was much higher than daidzein. Among the four constituents of *SR*, the content of baicalin was the highest, followed by wogonoside, both were much higher than baicalein and wogonin, respectively. Among the seven constituents of *CR*, the content of berberine was the highest, followed by several other alkaloids such as epiberberine, and the content of demethyleneberberine was much lower than above alkaloids. In terms of the *GR*, the content of glycyrrhizic acid was the highest, followed by liquiritin and isoliquiritin, while glycyrrhetinic acid was not detected.

**TABLE 2:** Contents of 17 constituents in the dried powder of *Gegen*-*Qinlian* decoction extract (mean ± SD, *n* = 3).

Compounds	Content (mg/g powder)		
Baicalin	59.87	±	6.30
Puerarin	43.60	±	0.60
Berberine	17.51	±	1.48
Daidzin	15.15	±	0.99
Wogonoside	11.12	±	0.81
Glycyrrhizic acid	9.07	±	0.33
Epiberberine	7.30	±	0.64
Coptisine	6.22	±	0.32
Palmatine	5.38	±	0.49
Jatrorrhizine	3.69	±	0.14
Liquiritin	2.42	±	0.16
Magnoflorine	1.97	±	0.17
Baicalein	1.15	±	0.03
Daidzein	0.73	±	0.04
Isoliquiritin	0.61	±	0.06
Wogonin	0.33	±	0.03
Demethyleneberberine	0.24	±	0.02

### 3.4 Pharmacokinetics of the constituents of oral GQD in mice

#### 3.4.1 Pharmacokinetics of the constituents in the portal vein

**FIGURE 1:**
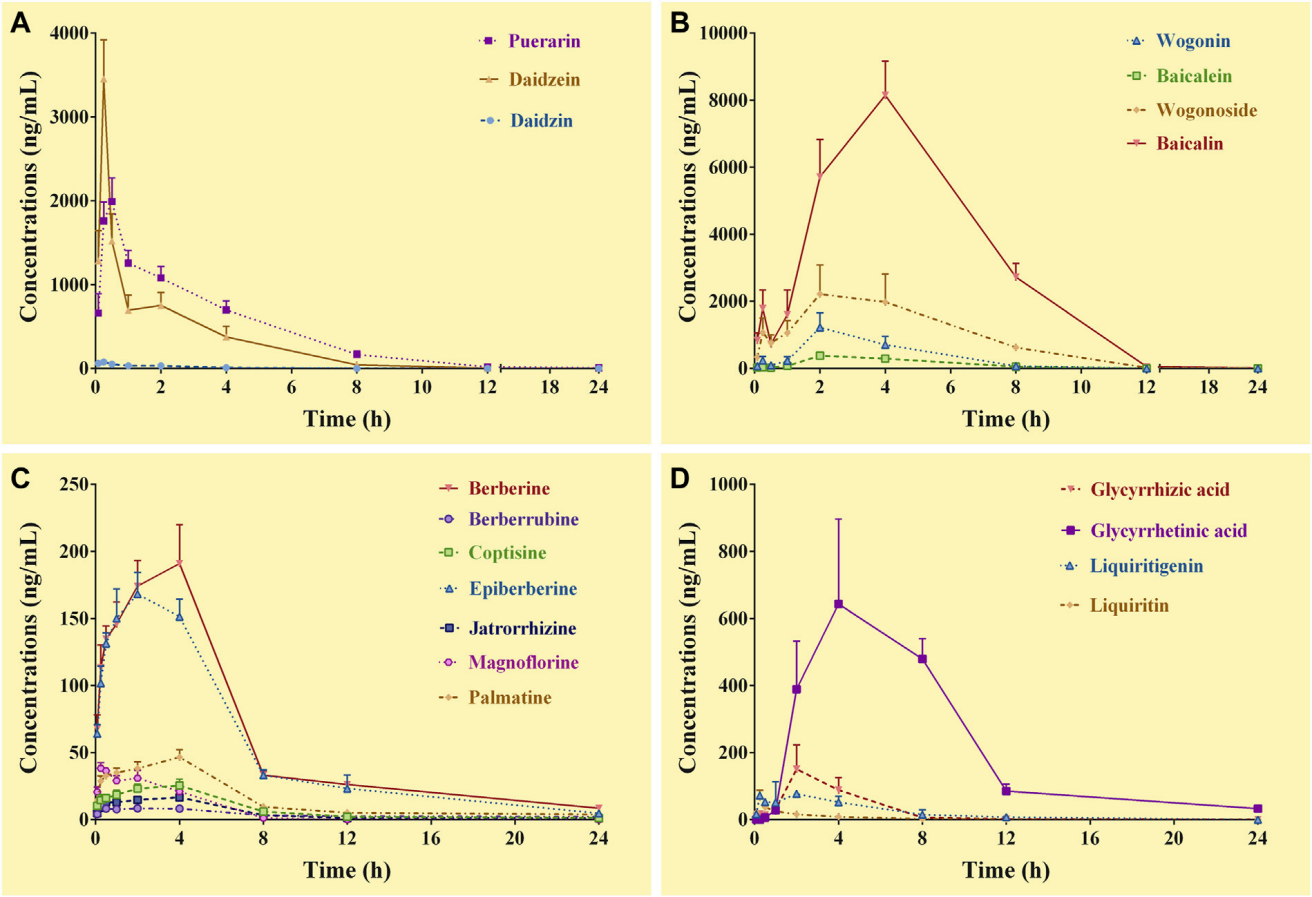
Concentration-time curves of the constituents of *Gegen*-*Qinlian* decoction in the portal vein of the mice received the oral *Gegen*-*Qinlian* decoction at the dosage of 6.1 g/kg (mean ± SD, n = 6). **(A)**, constituents derived from *Puerariae Lobatae Radix*; **(B)**, constituents derived from *Scutellariae Radix*; **(C)**, constituents derived from *Coptidis Rhizoma*; **(D)**, constituents derived from *Glycyrrhizae Radix Et Rhizoma Praeparata Cum Melle*.

The complete C-T curves of 18 GQD constituents (except demethyleneberberine) were obtained from the portal vein samples of the mice (Figure 1). The pharmacokinetic parameters were listed in Table 3. The results showed that daidzein and puerarin derived from *PR*, baicalin and wogonoside derived from *SR*, berberine and epiberberine derived from *CR*, glycyrrhetinic acid and liquiritigenin derived from *GR*, had relatively high exposure levels (C_max_ and AUC_0–24 h_) in the portal vein of the mice. These eight constituents could thus be considered as the major absorbed constituents of GQD after oral administration. Among these constituents, daidzein and puerarin were absorbed the fastest with T_max_ at 0.25–0.50 h; wogonoside, epiberberine, and liquiritigenin had T_max_ at 2.0 h, while baicalin, berberine, and glycyrrhetinic acid was the slowest in terms of absorption, with the T_max_ at 4.0 h. Among these constituents, berberine had the longest T_1/2_, which was at 8.0 h; the T_1/2_ of epiberberine, glycyrrhetinic acid, and liquiritigenin were within 4.0–6.0 h, while the T_1/2_ of puerarin, daidzein, baicalin, and wogonoside were within 2.0–3.0 h.

**TABLE 3:** Pharmacokinetic parameters of the constituents in the portal vein of the mice received oral *Gegen*-*Qinlian* decoction extract at the dosage of 6.1 g/kg (mean ± SD, n = 6).

Constituents	T_max_ (h)	C_max_ (ng/ml)	T_1/2_ (h)	AUC_0–24 h_ (h·ng/mL)	MRT (h)
Daidzin	0.25	76.9	1.8	151.0	2.0
Puerarin	0.50	1992.0	2.9	6709.5	3.2
Daidzein	0.25	3,455.2	2.6	4464.1	2.3
Baicalin	4.0	8146.7	2.0	46280.9	4.5
Wogonoside	2.0	2216.0	2.6	13370.9	4.1
Baicalein	2.0	381.0	1.3	1761.7	3.8
Wogonin	2.0	1223.3	1.0	4509.1	3.3
Berberine	4.0	190.9	8.0	1419.8	5.5
Epiberberine	2.0	168.2	5.6	1245.1	5.2
Palmatine	4.0	46.7	3.0	325.4	4.8
Coptisine	4.0	25.5	4.7	180.8	4.8
Jatrorrhizine	4.0	16.6	6.3	115.6	4.8
Magnoflorine	0.25	38.3	1.2	160.9	2.6
Berberrubine	0.25	10.3	5.1	67.0	4.4
Liquiritigenin	2.0	151.0	5.9	570.1	3.6
Liquiritin	0.25	66.9	2.5	110.1	2.5
Glycyrrhetinic acid	4.0	643.3	4.7	5334.4	7.0
Glycyrrhizic acid	2.0	77.1	2.9	422.1	3.7

#### 3.4.2 Pharmacokinetics of the constituents in the systemic circulation

**FIGURE 2:**
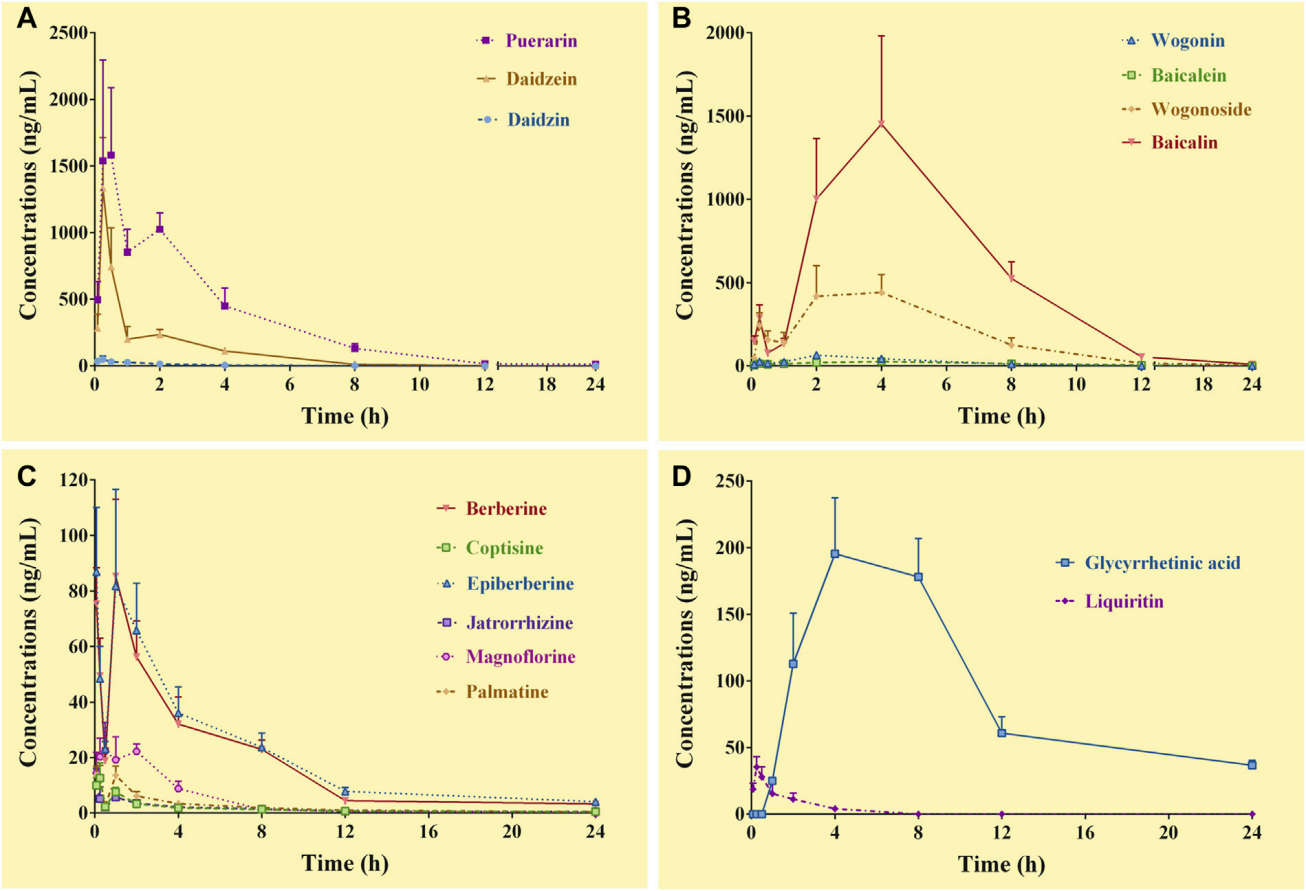
Concentration-time curves of the constituents of *Gegen*-*Qinlian* decoction in the systemic circulation of the mice received the oral *Gegen*-*Qinlian* decoction at the dosage of 6.1 g/kg (mean ± SD, n = 6). **(A)**, constituents derived from *Puerariae Lobatae Radix*; **(B)**, constituents derived from *Scutellariae Radix*; **(C)**, constituents derived from *Coptidis Rhizoma*; **(D)**, constituents derived from *Glycyrrhizae Radix Et Rhizoma Praeparata Cum Melle*.

Except for demethyleneberberine, berberrubine, liquiritigenin, and glycyrrhizic acid, the complete C-T curves of 15 GQD constituents were obtained in the systemic circulation (Figure 2). The pharmacokinetic parameters of the constituents were listed in Table 4. The results showed that puerarin and daidzein derived from *PR*, baicalin and wogonoside derived from *SR*, berberine and epiberberine derived from *CR*, and glycyrrhetinic acid derived from *GR* had relatively high exposure levels in the systemic circulation of the mice. Therefore, these seven constituents were the major GQD constituents entering the systemic circulation. Among these constituents, daidzein and puerarin were absorbed the fastest with T_max_ at 0.25–0.50 h; the T_max_ of berberine was 1.0 h; the T_max_ of epiberberine was 0.08 h, but actually the C-T curve of epiberberine showed double peak, with the time to reach the second peak at 1.0 h; the T_max_ of baicalin, wogonoside and glycyrrhetinic acid was all at 4.0 h. Among these constituents, the T_1/2_ of daidzein is 1.50 h, the T_1/2_ of puerarin, baicalin, and wogonoside was within 3.0–4.0 h, while glycyrrhetinic acid, berberine, and epiberberine were all about 6.0 h.

**TABLE 4:** Pharmacokinetic parameters of the constituents in the systemic circulation of the mice received oral *Gegen*-*Qinlian* decoction at the dosage of 6.1 g/kg (mean ± SD, n = 6).

Constituents	T_max_ (h)	C_max_ (ng/ml)	T_1/2_ (h)	AUC_0–24 h_ (h·ng/mL)	MRT (h)
Daidzin	0.25	49.0	1.8	85.7	1.8
Puerarin	0.50	1581.3	3.4	5234.4	3.3
Daidzein	0.25	1335.6	1.5	1482.5	1.9
Baicalin	4.0	1452.0	3.1	8667.1	5.1
Wogonoside	4.0	441.2	3.9	2830.4	4.6
Baicalein	4.0	26.7	6.3	228.6	6.8
Wogonin	2.0	64.7	1.5	300.4	3.6
Berberine	1.0	85.4	5.3	419.4	5.4
Epiberberine	0.08	86.9	5.3	479.8	5.8
Palmatine	1.0	13.7	7.2	53.3	5.5
Coptisine	0.25	12.6	10.8	35.2	6.4
Jatrorrhizine	0.08	10.1	4.3	26.3	3.9
Magnoflorine	0.50	22.4	1.9	94.9	2.6
Liquiritin	0.25	35.4	1.5	52.9	1.3
Glycyrrhetinic acid	4.0	195.5	6.2#	2122.9	8.4

#.the parameter might be inaccurately calculated due to incomplete elimination of the constituents.

#### 3.4.3 Pharmacokinetics of the constituents in the livers

**FIGURE 3:**
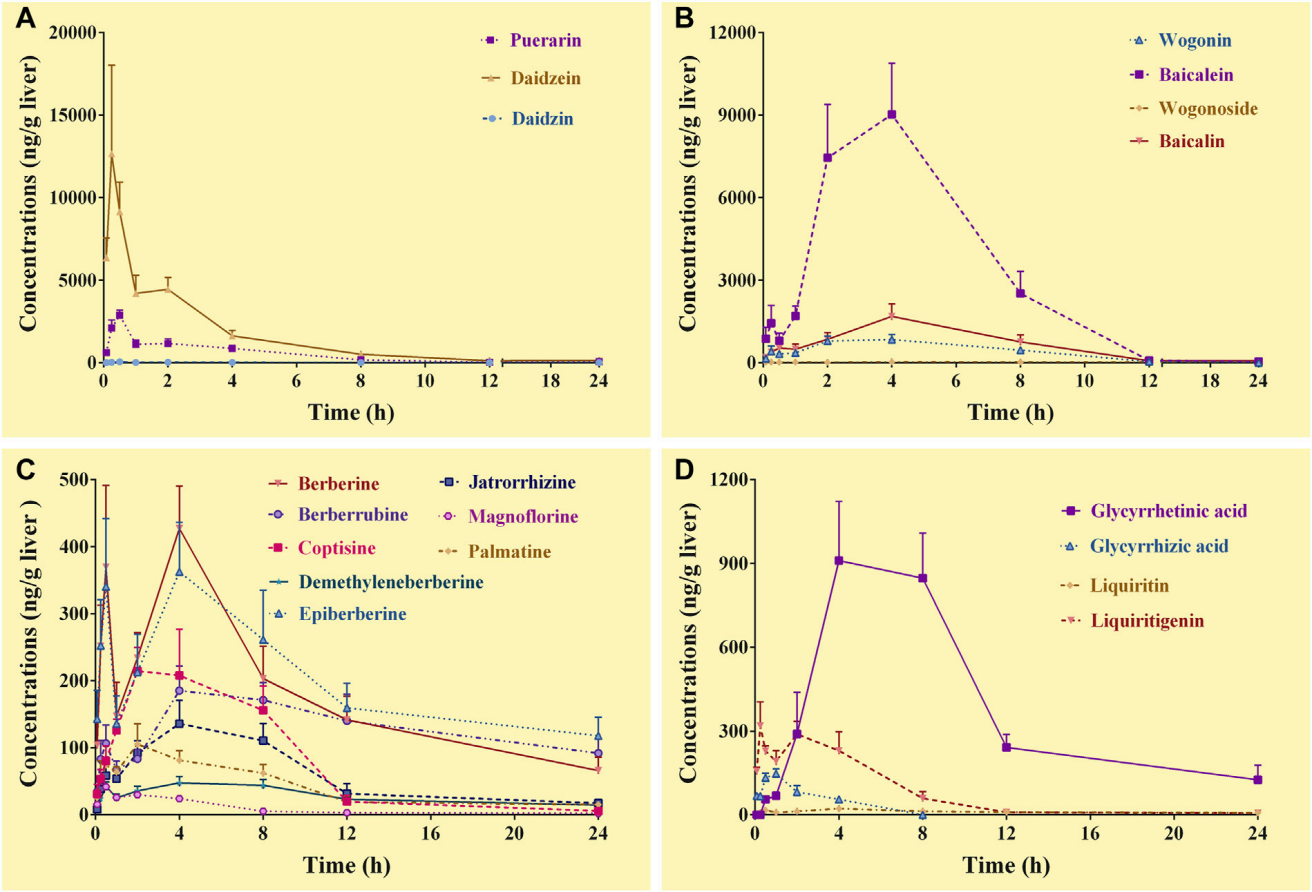
Concentration-time curves of the constituents of *Gegen*-*Qinlian* decoction in the livers of the mice received the oral *Gegen*-*Qinlian* decoction at the dosage of 6.1 g/kg (mean ± SD, n = 6). **(A)**, constituents derived from *Puerariae Lobatae Radix*; **(B)**, constituents derived from *Scutellariae Radix*; **(C)**, constituents derived from *Coptidis Rhizoma*; **(D)**, constituents derived from *Glycyrrhizae Radix Et Rhizoma Praeparata Cum Melle*.

Complete C-T curves of 19 GQD constituents were obtained in the liver samples (Figure 3). The pharmacokinetic parameters of the constituents were shown in Table 5. The results showed that puerarin and daidzein derived from *PR*, baicalein, baicalin and wogonin derived from *SR*, berberine, epiberberine, and berberrubine derived from *CR*, glycyrrhetinic acid and liquiritigenin derived from *GR* had relatively high exposure levels in the livers of the mice. Therefore, these ten constituents were the major GQD constituents distributed in the livers. Among these constituents, daidzein, puerarin, and liquiritigenin were absorbed the fastest with T_max_ at 0.25–0.50 h; the T_max_ of baicalin was 2.0 h; the T_max_ of baicalein, wogonin, berberine, epiberberine, berberrubine and glycyrrhetinic acid was all 4.0 h. In general, the T_1/2_ values of the GQD constituents in the livers were quite different. The constituents of *SR* had the shortest T_1/2_: baicalin was 1.0–2.0 h, baicalein and wogonin were 2.0–3.0 h; the T_1/2_ of the constituents of *PR* were longer than that of *SR*: puerarin and daidzein have T_1/2_ within 3.0–4.0 h; the T_1/2_ of liquiritigenin was 3.0–4.0 h, glycyrrhetinic acid was 6.0–7.0 h; the constituents of *CR* had the longest T_1/2_: berberine was about 10.0 h, while epiberberine and berberrubine were up to 15.0–19.0 h.

**TABLE 5:** Pharmacokinetic parameters of the constituents in the livers of the mice received the oral *Gegen*-*Qinlian* decoction extract at the dosage of 6.1 g/kg (mean ± SD, n = 6).

Constituents	T_max_ (h)	C_max_ (ng/g liver)	T_1/2_ (h)	AUC_0–24 h_ (ng·h/g liver)	MRT (h)
Daidzin	0.50	54.1	3.4	166.4	3.6
Puerarin	0.50	2871.7	4.0	8082.1	3.8
Daidzein	0.25	12,660.3	3.6	25330.7	3.3
Baicalin	2.0	5713.3	1.7	17447.0	3.7
Wogonoside	4.0	28.5	16.2	424.5	10.3
Baicalein	4.0	9020.0	2.3	51,101.1	4.3
Wogonin	4.0	838.9	2.6	6362.8	5.0
Berberine	4.0	427.2	10.1#	4291.2	8.3
Epiberberine	4.0	362.7	15.7#	4738.2	9.7
Palmatine	2.0	105.5	4.6	921.7	6.3
Coptisine	2.0	214.4	3.7	1901.4	5.8
Demethyleneberberine	4.0	47.7	11.1	685.2	9.4
Jatrorrhizine	4.0	136.2	2.4	1320.3	6.6
Magnoflorine	0.25	53.4	5.3	224.1	5.3
Berberrubine	4.0	185.5	18.2#	3152.5	11.0
Liquiritigenin	0.25	318.8	3.9	1783.9	4.2
Liquiritin	4.0	22.5	19.7	268.4	9.8
Glycyrrhetinic acid	4.0	910.1	6.6#	9306.5	8.6
Glycyrrhizic acid	1.0	147.5	3.8	526.0	2.8

#, the parameters might be inaccurately calculated due to incomplete elimination of the constituents.

#### 3.4.4 Pharmacokinetics of the constituents in the colons

**FIGURE 4:**
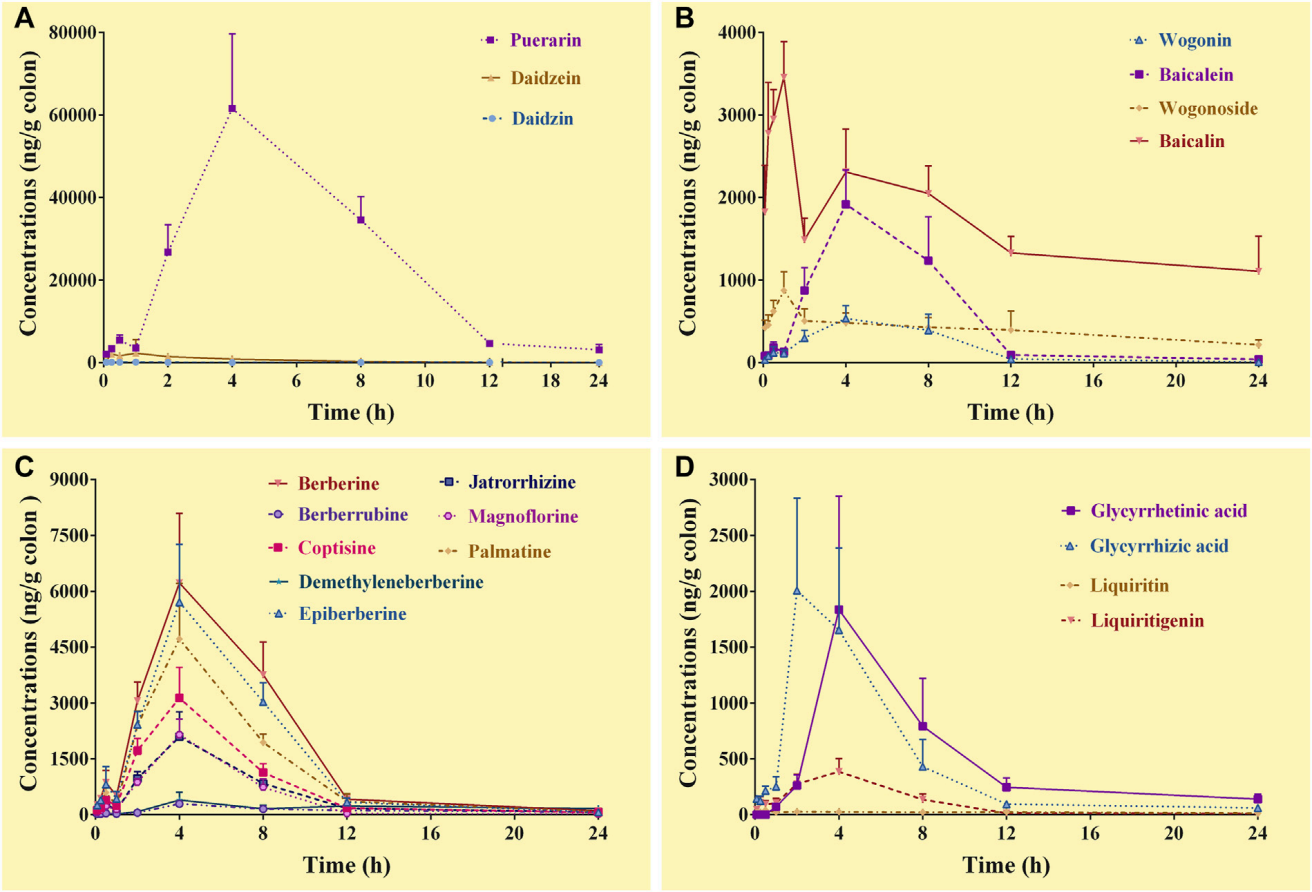
Concentration-time curves of the constituents of *Gegen*-*Qinlian* decoction in the colons of the mice received the oral *Gegen*-*Qinlian* decoction at the dosage of 6.1 g/kg (mean ± SD, n = 6). **(A)**, constituents derived from *Puerariae Lobatae Radix*; **(B)**, constituents derived from *Scutellariae Radix*; **(C)**, constituents derived from *Coptidis Rhizoma*; **(D)**, constituents derived from *Glycyrrhizae Radix Et Rhizoma Praeparata Cum Melle*.

In the colon samples, complete C-T curves of all the 19 GQD constituents were obtained (Figure 4). The pharmacokinetic parameters of the constituents were listed in Table 6. The results showed that puerarin and daidzein derived from *PR*, baicalin, baicalein and wogonoside derived from *SR*, berberine, epiberberine, palmatine, coptisine, jatrorrhizine, and magnoflorine derived from *CR*, glycyrrhetinic acid and glycyrrhizic acid derived from *GR* had relatively high exposure levels in the colons of the mice. Therefore, these thirteen constituents were the major constituents of GQD distributed in the colons. Among these constituents, daidzein, baicalin, and wogonoside were absorbed the fastest with T_max_ at 1.0 h; the T_max_ of glycyrrhizic acid was 2.0 h; the T_max_ of puerarin, baicalein, berberine, epiberberine, palmatine, coptisine, jatrorrhizine, magnoflorine and glycyrrhetinic acid were all at 4.0 h. In general, the T_1/2_ values of the GQD constituents in the colons were quite different. The constituents of *PR* had short T_1/2_: the T_1/2_ of puerarin and daidzein were within 1.0–4.0 h. The T_1/2_ of baicalin and wogonoside were up to 15.0–19.0 h, but baicalein and wogonin were within 2.0–3.0 h. The T_1/2_ of the alkaloid constituents derived from *CR* were mostly within 3.0–5.0 h. The T_1/2_ of glycyrrhizic acid was within 3.0–4.0 h, while the T_1/2_ of glycyrrhetinic acid was within 4.0–5.0 h.

**TABLE 6:** Pharmacokinetic parameters of the constituents in the colons of the mice received the oral *Gegen*-*Qinlian* decoction extract at the dosage of 6.1 g/kg (mean ± SD, n = 6).

Constituents	T_max_ (h)	C_max_ (ng/g colon)	T_1/2_ (h)	AUC_0–24 h_ (ng·h/g colon)	MRT (h)
Daidzin	1.0	113.6	11.0	1083.5	9.9
Puerarin	4.0	61585.3	1.3	406163.6	5.9
Daidzein	1.0	2293.1	3.5	9114.6	3.5
Baicalin	1.0	3457.7	18.6#	39145.1	9.9
Wogonoside	1.0	876.3	15.5#	9412.3	9.7
Baicalein	4.0	1917.5	2.6	13011.6	5.8
Wogonin	4.0	534.8	3.2	4182.0	6.1
Berberine	4.0	6226.0	3.5	43093.5	6.0
Epiberberine	4.0	5705.3	3.3	36717.4	5.9
Palmatine	4.0	4719.0	3.8	29379.3	5.9
Coptisine	4.0	3136.5	4.3	18699.5	5.6
Demethyleneberberine	4.0	395.1	2.4	3847.0	8.5
Jatrorrhizine	4.0	2096.6	5.0	13120.3	6.2
Magnoflorine	4.0	2152.3	2.1	11227.0	5.0
Berberrubine	4.0	293.1	14.3	2971.2	10.1
Liquiritigenin	4.0	386.0	3.4	2380.9	4.9
Liquiritin	0.50	41.2	19.5	504.2	10.1
Glycyrrhetinic acid	4.0	1834.8	4.4	11419.8	7.0
Glycyrrhizic acid	2.0	2006.4	3.3	10890.5	4.8

#, the parameters might be inaccurately calculated due to incomplete elimination of the constituents.

#### 3.4.5 Comparison of the AUC values

As shown in Table 7, the AUC values of some GQD constituents in the portal vein, the livers, and the systemic circulation were significantly different. The concentration of wogonoside in the livers was much lower than that in the portal vein or the systemic circulation. However, the concentrations of daidzein, baicalein, wogonin, berberine, palmatine, coptisine, and jatrorrhizine in the livers were more than 10 times higher than those in the systemic circulation; the concentrations of liquiritin and glycyrrhetinic acid in the livers were about 5 times higher than those in the systemic circulation; in addition, demethyleneberberine, berberrubine, liquiritigenin, and glycyrrhizic acid were detected in the livers but not in the systemic circulation. The above results suggested that these constituents were accumulated in the livers.

**TABLE 7:** Comparison of the AUC_0–24 h_ values of the major constituents of *Gegen*-*Qinlian* decoction in the livers (AUC_0–24 h liv)_ with those in the blood of portal vein (AUC_0–24 h por_) or systemic circulation (AUC_0–24 h cir_).

Constituents	AUC_0–24 h liv_/AUC_0–24 h por_	AUC_0–24 h liv_/AUC_0–24 h cir_
Daidzin	1.1	1.9
Puerarin	1.2	1.5
Daidzein	5.7	17.1
Baicalin	0.4	2.0
Wogonoside	0.03	0.15
Baicalein	29.0	223.6
Wogonin	1.4	21.2
Berberine	3.0	10.2
Epiberberine	3.8	9.9
Palmatine	2.8	17.3
Coptisine	10.5	54.0
Demethyleneberberine	NC	NC
Jatrorrhizine	11.4	50.2
Magnoflorine	1.4	2.4
Berberrubine	47.1	NC
Liquiritigenin	3.1	NC
Liquiritin	2.4	5.1
Glycyrrhetinic acid	1.7	4.4
Glycyrrhizic acid	1.2	NC

NC, the ratio was not calculated because the constituent was only detected in the livers but not in the portal vein or systemic circulation of the mice.

#### 3.4.6 Integrated pharmacokinetics of the GQD constituents

**FIGURE 5:**
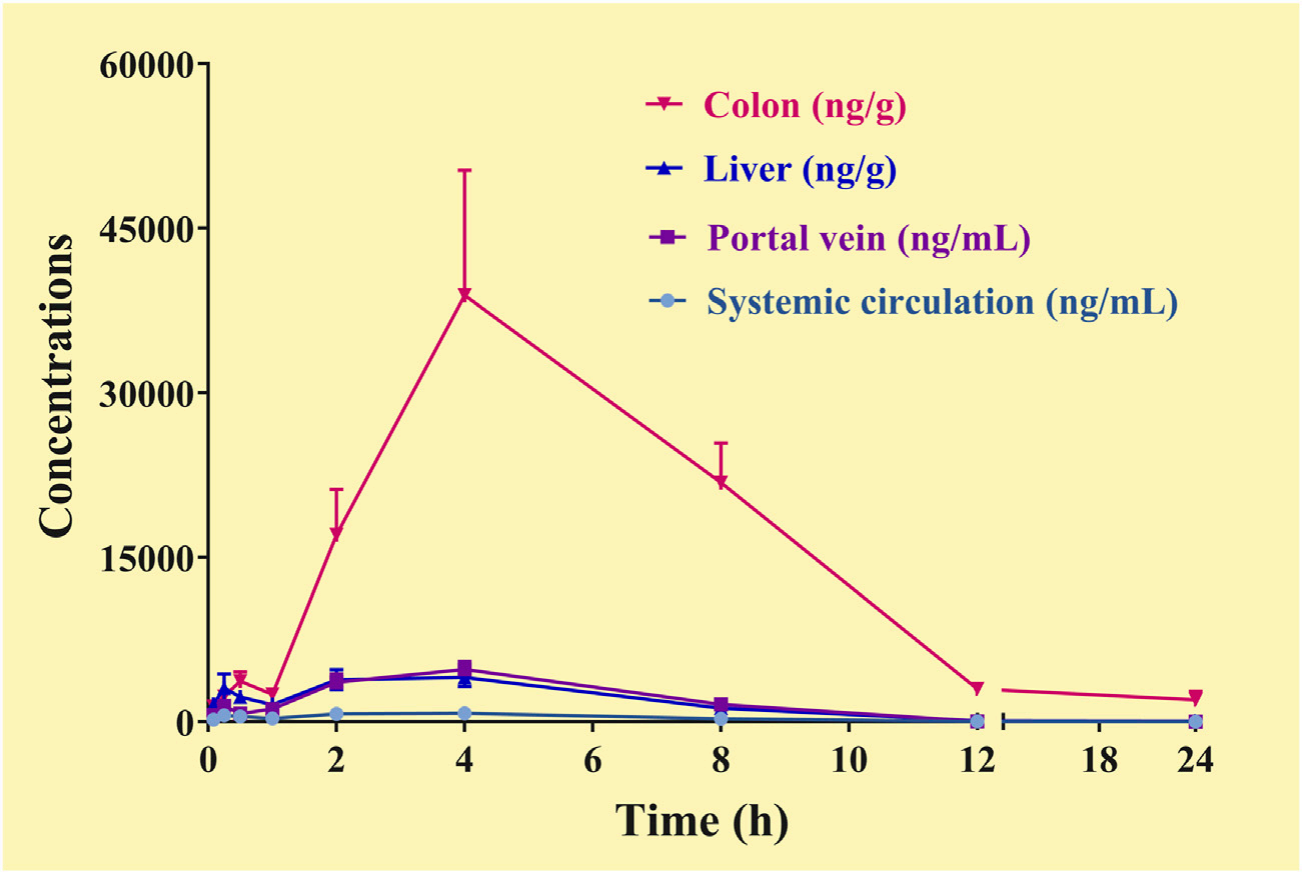
Concentration-time curve of the “integrated constituent” in the plasma (the systemic circulation, the portal vein) and tissue (the liver, the colon) samples of the mice received the oral *Gegen*-*Qinlian* decoction at the dosage of 6.1 g/kg (mean ± SD, n = 6).

The C-T curves of the GQD “integrated constituent” were shown in Figure 5, and the pharmacokinetic parameters were listed in Table 8. The GQD “integrated constituent” reached peak concentration at 4.0 h in the portal vein, the systemic circulation, the livers, and the colons, with half-lives of 1.5–4.1 h and mean retention time of 4.5–6.3 h, respectively. The concentrations of the “integrated constituent” in the livers and the colons were much higher than that in the systemic circulation, indicating its accumulation in these tissues. Furthermore, the concentration of the GQD “integrated constituent” in the colons was approximately 10 times higher than in the livers, suggesting that the colon might be a more important target tissue for GQD than the liver.

**TABLE 8:** Pharmacokinetic parameters of the “integrated constituent” in the blood or tissue samples of the mice received the oral *Gegen*-*Qinlian* decoction extract at the dosage of 6.1 g/kg.

Samples	T_max_ (h)	C_max_# (ng/ml)	T_1/2_ (h)	AUC_0–24 h_* (h·ng/mL)	MRT (h)
Systemic circulation	4.0	760.7	3.1	5300.6	4.6
Portal vein	4.0	4788.5	2.3	28015.1	4.5
Liver	4.0	4040.0	1.5	26820.6	4.5
Colon	4.0	38886.8	4.1	263328.7	6.3

#, the unit is “ng/g” for the liver and colon samples; *, the unit is “h·ng/g” for the liver and colon samples.

## 4 Discussion

The LC-MS/MS method established in this study could be used to simultaneously and quantitatively detect the concentrations of 19 GQD constituents in the plasma and tissue homogenate samples. The method had no endogenous interference and therefore had good specificity. The method had high sensitivity. The LLOQ of the constituents met the requirements of pharmacokinetic studies, that is, the constituent in the samples obtained at least three to five T_1/2_ after oral administration or the constituent with concentration that were 1/10-1/20 of its C_max_ could be determined. In addition, the items including intraday and interday precision and accuracy, recovery, matrix effect and stability of each constituent were good, and there was no significant residue of the constituents. Pharmacokinetic analysis showed that the concentrations of most constituents were within their linear ranges of the established method. Because the method had good dilution linearity, individual samples with concentrations higher than the ULOQs could also be accurately determined after dilution. The previously reported methods could be used to simultaneously measure the concentrations of only 8-12 GQD constituents (Zhang et al., 2014; Xu B. L. et al., 2015; Zhang et al., 2015; Ling et al., 2017). An excellent reported LC-MS/MS method allowed simultaneous measurement of the concentrations of 42 GQD constituents (Qiao et al., 2018). However, the method was developed to analyze plasma samples and its applicability to tissue samples is unclear.

Puerarin, daidzein and daidzein are representative constituents of *PR*. Puerarin was the most exposed constituents of GQD in the colons, which reached concentration as high as 61,585.3 ng/g in the colons. In addition, the concentration of puerarin in the colons was much higher than that in the portal vein (1992.0 ng/ml), the systemic circulation (1581.3 ng/ml) and the livers (2871.7 ng/g), suggesting that the colon might be the most important target tissue of puerarin. Puerarin is extensively metabolized into 66 metabolites after oral absorption (Shang et al., 2017), explaining why the concentrations of puerarin in portal vein, liver and systemic circulation were significantly lower than that in the colons. *In vivo* experiments have confirmed that oral administration of 10 or 50 mg/kg puerarin alleviated dextran sodium sulfate-induced colitis due to anti-inflammatory, antioxidant, and intestinal epithelial cell barrier improving effects (Jeon et al., 2020). In addition, oral administration of 6–24 mg/kg puerarin improved symptoms of rats with irritable bowel syndrome, promoted colonic epithelial cell proliferation and repaired colonic mucosal barrier of the rats (Wang et al., 2021). The content of daidzin was much higher than the content of daidzein in GQD extract, while the concentration of daidzein was significantly higher than the concentration of daidzin in portal vein. It was reported that in the process of intestinal absorption, most of daidzin could be metabolized by intestinal β-glucosidases (Setchell et al., 2002). This was the reason why daidzin had the highest concentration in the colons. The concentration of daidzein in the livers reached a concentration as high as 12,660.3 ng/g, which is beneficial for daidzein to regulate glycolipid metabolism in the liver (Das et al., 2018) and protect the liver from injury (Yu et al., 2020). Daidzein was enriched in the livers because it is a major metabolite of both puerarin (Shang et al., 2017) and daidzein (Wilkinson et al., 2003).

Baicalin, wogonoside, baicalein, and wogonin are the representative constituents of *SR*. In this study, the highest concentration of baicalin in the livers reached 5713.3 ng/g, and the highest concentration in the colons reached 3,457.7 ng/g, suggesting that both the liver and the colon could be important target tissues of baicalin. Studies have confirmed that baicalin has the effects of protecting hepatic injury (Shi L. et al., 2018), improving hepatic steatosis (Dai et al., 2018), and relieving ulcerative colitis (Shen et al., 2019). Interestingly, the concentration of baicalin in the portal vein was even higher than those in the colons and the livers. This is because a portion of baicalein could be metabolized to baicalin after absorption and then released into the portal vein (Akao et al., 2000). The highest concentration of baicalein in the livers reached 9020.0 ng/g, which is beneficial for its curative effects on liver injury (Dai et al., 2021) and liver fat accumulation (Sun et al., 2020). Baicalin can be metabolized to baicalein by the liver (Liu et al., 2008), resulting in accumulation of baicalein in the livers. It also had a high exposure level (1917.5 ng/g) in the colons, so it could also play an anti-ulcerative colitis role in the colon (Li et al., 2021). It is worth noting that the concentration of baicalin in the systemic circulation was 1452.0 ng/ml, which was lower than its highest concentration in the livers and the portal vein (8,146.7 ng/ml); while the highest concentration of baicalein in the systemic circulation (26.7 ng/ml) was far below its highest concentration in the livers and the portal vein (381.0 ng/ml). It has long been found that baicalin can be hydrolyzed by intestinal bacteria into baicalein, absorbed in the form of baicalein, and then metabolized into baicalin in intestinal epithelial cells or the liver and then enter the systemic circulation (Akao et al., 2000; Zhang et al., 2005; Zhang et al., 2007). The results may explain why the concentration of baicalin in the portal vein, the systemic circulation, and the colons were higher than that of baicalein. On one hand, baicalin can be metabolized to baicalein (Liu et al., 2008); on the other hand, baicalin can be actively excreted into the bile by the efflux transporter including multidrug resistance-associated protein (MRP) and breast cancer resistance protein (BCRP) (Zhang L. et al., 2011), explaining why the concentration of baicalin in the livers was lower than that of baicalein. In general, the concentrations of wogonoside and wogonin in the livers and the colons were much lower than those of baicalin and baicalein, therefore, their roles in GQD were not as significant as those of baicalin and baicalein.

Alkaloids such as berberine, epiberberine, coptisine, palmatine, jatrorrhizine, desmethyleneberberine, berberrubine, and magnoflorine are representative constituents of *CR*. The concentrations of each alkaloid in the portal vein and the systemic circulation were low, but they were accumulated significantly in the livers and the colons. Taking berberine as an example, its highest concentrations in the portal vein and the systemic circulation were 190.9 ng/ml and 85.4 ng/ml, respectively, but reached 427.2 ng/g and 6226.0 ng/g in the livers and the colons, respectively. Berberine is a common substrate of both organic cation transporter and organic anion-transporting polypeptide (Chen et al., 2015), which are highly expressed in the intestine and the liver (Roth et al., 2012). In addition, berberine accumulates in cells due to mitochondrial membrane potential (Li et al., 2019). These mechanisms might contribute to the enrichment of berberine as well as other alkaloids in the colons and the livers. After oral administration of *CR* or berberine, the exposure level (AUC) of berberine in the livers can reach more than 200 times the exposure level in the systemic circulation (Li et al., 2018; Zhao et al., 2021a; Zhao et al., 2021b). In this study, the ratio was only about 12 times, probably because the liver distribution of berberine was reduced by the interaction among GQD constituents. In general, after GQD was orally administered, the concentration of the alkaloids in the livers was too low to exert their potential pharmacological effects of protecting the liver from injury and regulating glucose and lipid metabolism in the liver (Bansod et al., 2021). In contrast, in addition to berberine, epiberberine (5705.3 ng/g), coptisine (3,136.5 ng/g), palmatine (4719.0 ng/g), jatrorrhizine (2096.6 ng/g), and magnoflorine (2152.3 ng/g) had very high concentrations in the colons. Studies have shown that berberine has significant anti-inflammatory effect at the concentration of 10 μM (Zhai et al., 2020), and *in vivo* experiments have proved that oral administration of 40 mg/kg berberine for 7 days significantly improved intestinal mucosal barrier function and reduce inflammatory reaction in dextran sodium sulfate-induced ulcerative colitis model mice (Dong et al., 2022), suggesting that the colon might be the major target tissue for the alkaloid constituents derived from *CR* after oral administration of GQD.

Glycyrrhizic acid, glycyrrhetinic acid, liquiritin, and liquiritigenin are the representative constituents of *GR*. Glycyrrhetinic acid was the most exposed *GR* constituents after oral administration of GQD. The highest concentration of glycyrrhetinic acid in the livers (910.1 ng/g) and the colons (1834.8 ng/g) was higher than that in the systemic circulation (195.5 ng/ml), indicating that glycyrrhetinic acid was accumulated in the livers and the colons. Studies have shown that there are glycyrrhetinic acid-specific receptors on hepatocytes (Stecanella et al., 2021), which may be related to their accumulation in the livers; however, it is not clear whether glycyrrhetinic acid receptors are also expressed in the colon. Thus, the liver and the colon became important target tissues of glycyrrhetinic acid. Studies confirmed that glycyrrhetinic acid has hepatoprotective (Wu et al., 2021) and choleretic (Yan et al., 2021) effects. Glycyrrhizic acid was the highest *GR* constituent in GQD, but the concentration of glycyrrhizic acid in the portal vein, the systemic circulation, and the livers was relatively low, because most of glycyrrhizic acid could be metabolized by intestinal bacteria into glycyrrhetinic acid (Kim et al., 2000). Surprisingly, the concentration of glycyrrhizic acid in the colons still reached 2006.4 ng/g, being beneficial for its anti-ulcerative colitis effect (Hu et al., 2022).

Integrated pharmacokinetic studies showed that the GQD “integrated constituent” reached peak concentration at 4.0 h in the portal vein, the systemic circulation, the livers, and the colons, with half-lives of 1.5–4.1 h and mean retention time of 4.5–6.3 h, respectively. In addition, the constituents of GQD were accumulated in the livers and the colons, which were associated with the pharmacological effects of GQD: it exerts systemic/absorptive effects on reducing blood sugar and lipid absorption in the liver, and it has a local effect on intestinal diseases in the colon. Furthermore, the concentration of the “integrated constituent” in the colons was much higher than that in the livers, suggesting that inflammatory intestinal diseases is likely to be the main indication of oral GQD.

A deficiency of the pharmacokinetic study was that after 24 h of oral administration of GQD, some constituents in the colons and the livers were not completely eliminated, which might affect the calculation of their T_1/2_ values in the tissues. However, the decline in the C-T curves of the “integrated constituent” was obvious. Therefore, the influence on the calculation of the T_1/2_ values of the “integrated constituent” was limited. The integrated pharmacokinetics of GQD in rat plasma has been reported (Zhang Q.-Y. et al., 2011). However, it should be pointed out that in this study, only the complete C-T curves of eight GQD constituents were obtained, while the major constituents including puerarin, daidzein, glycyrrhetinic acid, and epiberberine were not included in the study. In addition, compared to other studies, the T1/2 values of the alkaloids were significantly longer without reasonable explanations [25.8–29.0 h (Zhang Q.-Y. et al., 2011) vs*.* 1.6–11.4 h (Qiao et al., 2018) or 7.0–7.5 h (Zhang et al., 2014)].

Integrated pharmacokinetics was proposed in 2008 and it promoted the studies on the pharmacokinetic properties of multicomponent complexes such as TCMs (Li et al., 2008; Hao et al., 2009; Zhu et al., 2012; He et al., 2014; Shi P. et al., 2018). However, it should be noted that this strategy do not distinguish the efficacy of each constituent. Therefore, in addition to AUC values, it is recommended to apply pharmacodynamic weighting coefficients when conducting integrated pharmacokinetic studies. Since a TCM often has multiple pharmacological effects, it may be necessary to integrate specific C-T curves according to specific pharmacological effects. Certainly, this modified strategy may still be challenging for complex diseases, such as intestinal and liver diseases in which GQD is effective.

## 5 Conclusion

The established method was quick, sensitive, and accurate enough to analyze GQD constituents in plasma and tissue homogenate samples quantitatively. The entire C-T curves of 18, 15, 19, and 19 constituents were respectively obtained from the portal vein, systemic circulation, liver, and colon samples of the mice after oral administration of GQD. Daidzein, baicalin, and baicalein had relatively high exposure levels in the livers, while puerarin, berberine, epiberberine, coptisine, palmatine, jatrorrhizine, magnoflorine, glycyrrhizic acid, and glycyrrhetinic acid were enriched in the colons. Given that these constituents have significant biological activity, they could be regarded as the major effective constituents of GQD in treating colon or liver-related diseases, respectively. In addition, the integrated pharmacokinetic properties of GQD were studied. The GQD “integrated constituent” reached peak concentration at 4.0 h in the portal vein, the systemic circulation, the livers, and the colons, with half-lives of 1.5–4.1 h and mean retention time of 4.5–6.3 h, respectively. Furthermore, the concentration of the GQD “integrated constituent” in the colons was approximately 10 times higher than that in the livers, both of which were much higher than that in the systemic circulation, indicating its accumulation in these tissues, especially in the colons.

In conclusion, the tissue distribution and integrated pharmacokinetic properties of major effective constituents of oral GQD in mice were revealed in this study. The results of the tissue distribution study would contribute to identifying the major target tissues and effective constituents of GQD, while the results of the integrated pharmacokinetic study would help to explain the pharmacokinetic properties of oral GQD as a whole.

## Data Availability

The original contributions presented in the study are included in the article/Supplementary Material, further inquiries can be directed to the corresponding authors.

## Abbreviations

AUC, area under the concentration-time curve; Cmax, peak concentration; CR, Coptidis Rhizoma; C-T, concentration-time; GQD, Gegen-Qinlian decoction; GR, Glycyrrhizae Radix et Rhizoma Praeparata Cum Melle; IS, internal standard; LC-MS/MS, liquid chromatography-tandem mass spectrometry; LLOQ, lower limit of quantification; MRT, mean retention time; PR, Pueraria Lobatae Radix; QC, quality control; SR, Scutellaria Radix; T1/2, half-life; TCM, traditional Chinese medicine; Tmax, peak concentration; ULOQ, upper limit of quantification.

## References

[B1] Akao T. Kawabata K. Yanagisawa E. Ishihara K. Mizuhara Y. Wakui Y. (2000). Baicalin, the predominant flavone glucuronide of scutellariae radix, is absorbed from the rat gastrointestinal tract as the aglycone and restored to its original form. J. Pharm. Pharmacol. 52, 1563–1568. 10.1211/0022357001777621 11197087

[B2] Bansod S. Saifi M. A. Godugu C. (2021). Molecular updates on berberine in liver diseases: Bench to bedside. Phytother. Res. 35, 5459–5476. 10.1002/ptr.7181 34056769

[B3] Cao Z. Zeng Z. Wang B. Liu C. Liu C. Wang Z. (2020). Identification of potential bioactive compounds and mechanisms of GegenQinlian decoction on improving insulin resistance in adipose, liver, and muscle tissue by integrating system pharmacology and bioinformatics analysis. J. Ethnopharmacol. 264, 113289. 10.1016/j.jep.2020.113289 32846191

[B4] Chen C. Wu Z.-T. Ma L.-L. Ni X. Lin Y.-F. Wang L. (2015). Organic anion-transporting polypeptides contribute to the hepatic uptake of berberine. Xenobiotica. 45, 1138–1146. 10.3109/00498254.2015.1042537 26068524

[B5] Dai C. Li H. Wang Y. Tang S. Velkov T. Shen J. (2021). Inhibition of oxidative stress and ALOX12 and NF-κB pathways contribute to the protective effect of baicalein on carbon tetrachloride-induced acute liver injury. Antioxidants (Basel) 10, 976. 10.3390/antiox10060976 34207230 PMC8235740

[B6] Dai J. Liang K. Zhao S. Jia W. Liu Y. Wu H. (2018). Chemoproteomics reveals baicalin activates hepatic CPT1 to ameliorate diet-induced obesity and hepatic steatosis. Proc. Natl. Acad. Sci. U. S. A. 115, E5896–E5905. 10.1073/pnas.1801745115 29891721 PMC6042128

[B7] Das D. Sarkar S. Bordoloi J. Wann S. B. Kalita J. Manna P. (2018). Daidzein, its effects on impaired glucose and lipid metabolism and vascular inflammation associated with type 2 diabetes. Biofactors 44, 407–417. 10.1002/biof.1439 30191623

[B8] Dong Y. Fan H. Zhang Z. Jiang F. Li M. Zhou H. (2022). Berberine ameliorates DSS-induced intestinal mucosal barrier dysfunction through microbiota-dependence and Wnt/β-catenin pathway. Int. J. Biol. Sci. 18, 1381–1397. 10.7150/ijbs.65476 35280677 PMC8898376

[B9] Hai C. X. Wei L. (2009). Gegen qinlian decoction. China Medical Science and Technology Press. Bei Jing

[B10] Hao H. P. Zheng C. N. Wang G. J. (2009). Thoughts and experimental exploration on pharmacokinetic study of herbal medicines with multiple-components and targets. Yao Xue Xue Bao 44, 270–275. 19449523

[B11] He W. Liu G. Cai H. Sun X. Hou W. Zhang P. (2014). Integrated pharmacokinetics of five protoberberine-type alkaloids in normal and insomnic rats after single and multiple oral administration of Jiao-Tai-Wan. J. Ethnopharmacol. 154, 635–644. 10.1016/j.jep.2014.04.040 24815220

[B12] Hu H. Lei Y. Zhang W. Xiong P. Song L. Luo X. (2022). Anti-inflammatory activity and safety of compound glycyrrhizin in ulcerative colitis: A systematic review and meta-analysis of randomized controlled trials. J. Funct. Foods 91, 105004. 10.1016/j.jff.2022.105004

[B13] Jeon Y. D. Lee J. H. Lee Y. M. Kim D. K. (2020). Puerarin inhibits inflammation and oxidative stress in dextran sulfate sodium-induced colitis mice model. Biomed. Pharmacother. 124, 109847. 10.1016/j.biopha.2020.109847 31981944

[B14] Kim D. H. Hong S. W. Kim B. T. Bae E. A. Park H. Y. Han M. J. (2000). Biotransformation of glycyrrhizin by human intestinal bacteria and its relation to biological activities. Arch. Pharm. Res. 23, 172–177. 10.1007/BF02975509 10836746

[B15] Li Q. Yang Y. Zhou T. Wang R. Li N. Zheng M. (2018). A Compositive Strategy to Study the Pharmacokinetics of TCMs: Taking Coptidis Rhizoma, and Coptidis Rhizoma-Glycyrrhizae Radix et Rhizoma as Examples. Mol. (Basel, Switz. 23, E2042. 10.3390/molecules23082042 PMC6222803 30111723

[B16] Li Q. Zhou T. Liu C. Wang X. Y. Zhang J. Q. Wu F. (2019). Mitochondrial membrane potential played crucial roles in the accumulation of berberine in HepG2 cells. Biosci. Rep. 39, BSR20190477. Artn Bsr20190477. 10.1042/Bsr20190477 30944202 PMC6487271

[B17] Li X.-Y. Hao H.-P. Wang G.-J. Sun J.-G. Liang Y. Xie L. (2008). Integrated pharmacokinetic study of multiple effective components contained in total panax notoginsenosides. Chin. J. Nat. Med. 6, 377–381.

[B18] Li Y. Y. Wang X. J. Su Y. L. Wang Q. Huang S. W. Pan Z. F. (2021). Baicalein ameliorates ulcerative colitis by improving intestinal epithelial barrier via AhR/IL-22 pathway in ILC3s. Acta Pharmacol. Sin. 43, 1495–1507. 10.1038/s41401-021-00781-710.1038/s41401-021-00781-7 34671110 PMC9160000

[B19] Ling X. Xiang Y. Q. Tang Q. F. Chen F. L. Tan X. M. (2017). Comparative pharmacokinetics of eight major bioactive components in normal and bacterial diarrhea mini-pigs after oral administration of Gegen Qinlian Decoction. J. Chromatogr. B Anal. Technol. Biomed. Life Sci. 1044, 132–141. 10.1016/j.jchromb.2017.01.015 28107700

[B20] Liu C. S. Liang X. Wei X. H. Jin Z. Chen F. L. Tang Q. F. (2019). Gegen qinlian decoction treats diarrhea in piglets by modulating gut microbiota and short-chain fatty acids. Front. Microbiol. 10, 825. ARTN 825. 10.3389/fmicb.2019.00825 31057525 PMC6482297

[B21] Liu T. Tian X. M. Li Z. Q. Han F. Ji B. Zhao Y. L. (2018). Metabolic profiling of Gegenqinlian decoction in rat plasma, urine, bile and feces after oral administration by ultra high performance liquid chromatography coupled with Fourier transform ion cyclotron resonance mass spectrometry. J. Chromatogr. B Anal. Technol. Biomed. Life Sci. 1079, 69–84. 10.1016/j.jchromb.2018.02.001 29453016

[B22] Liu Y. T. Hao H. P. Xie H. G. Lai L. Wang Q. Liu C. X. (2010). Extensive intestinal first-pass elimination and predominant hepatic distribution of berberine explain its low plasma levels in rats. Drug Metab. Dispos. 38, 1779–1784. [pii]. 10.1124/dmd.110.033936 20634337

[B23] Liu Z. M. Ma Y. M. Wang T. M. Guo X. (2008). *In vitro* metabolic interconversion between baicalin and baicalein in the liver, kidney, intestine and bladder of rat. Yaoxue Xuebao 43, 664–668. 18822973

[B24] Lu J. Z. Ye D. Ma B. L. (2021). Constituents, pharmacokinetics, and pharmacology of gegen-qinlian decoction. Front. Pharmacol. 12, 668418. 10.3389/fphar.2021.668418 34025427 PMC8139575

[B25] Qiao X. Wang Q. Wang S. Kuang Y. Li K. Song W. (2018). A 42-markers pharmacokinetic study reveals interactions of berberine and glycyrrhizic acid in the anti-diabetic Chinese medicine formula gegen-qinlian decoction. Front. Pharmacol. 9, 622. ARTN 622. 10.3389/fphar.2018.00622 29971002 PMC6018403

[B26] Roth M. Obaidat A. Hagenbuch B. (2012). OATPs, OATs and OCTs: The organic anion and cation transporters of the SLCO and SLC22A gene superfamilies. Br. J. Pharmacol. 165, 1260–1287. 10.1111/j.1476-5381.2011.01724.x 22013971 PMC3372714

[B27] Setchell K. D. Brown N. M. Zimmer-Nechemias L. Brashear W. T. Wolfe B. E. Kirschner A. S. (2002). Evidence for lack of absorption of soy isoflavone glycosides in humans, supporting the crucial role of intestinal metabolism for bioavailability. Am. J. Clin. Nutr. 76, 447–453. 10.1093/ajcn/76.2.447 12145021

[B28] Shang Z. Xin Q. Zhao W. Wang Z. Li Q. Zhang J. (2017). Rapid profiling and identification of puerarin metabolites in rat urine and plasma after oral administration by UHPLC-LTQ-Orbitrap mass spectrometer. J. Chromatogr. B Anal. Technol. Biomed. Life Sci. 1068-1069, 180–192. 10.1016/j.jchromb.2017.10.038 29073480

[B29] Shen J. Cheng J. Zhu S. Zhao J. Ye Q. Xu Y. (2019). Regulating effect of baicalin on IKK/IKB/NF-kB signaling pathway and apoptosis-related proteins in rats with ulcerative colitis. Int. Immunopharmacol. 73, 193–200. 10.1016/j.intimp.2019.04.052 31103874

[B30] Shi L. Hao Z. Zhang S. Wei M. Lu B. Wang Z. (2018a). Baicalein and baicalin alleviate acetaminophen-induced liver injury by activating Nrf2 antioxidative pathway: The involvement of ERK1/2 and PKC. Biochem. Pharmacol. 150, 9–23. 10.1016/j.bcp.2018.01.026 29338970

[B31] Shi P. Lin X. Yao H. (2018b). A comprehensive review of recent studies on pharmacokinetics of traditional Chinese medicines (2014-2017) and perspectives. Drug Metab. Rev. 50, 161–192. 10.1080/03602532.2017.1417424 29258334

[B32] Stecanella L. A. Bitencourt A. P. R. Vaz G. R. Quarta E. Silva Junior J. O. C. Rossi A. (2021). Glycyrrhizic acid and its hydrolyzed metabolite 18β-glycyrrhetinic acid as specific ligands for targeting nanosystems in the treatment of liver cancer. Pharmaceutics 13, 1792. 10.3390/pharmaceutics13111792 34834206 PMC8621092

[B33] Sun W. Liu P. Wang T. Wang X. Zheng W. Li J. (2020). Baicalein reduces hepatic fat accumulation by activating AMPK in oleic acid-induced HepG2 cells and high-fat diet-induced non-insulin-resistant mice. Food Funct. 11, 711–721. 10.1039/c9fo02237f 31909773

[B34] Wang Q. S. Wang Y. L. Zhang W. Y. Li K. D. Luo X. F. Cui Y. L. (2021). Puerarin from Pueraria lobata alleviates the symptoms of irritable bowel syndrome-diarrhea. Food Funct. 12, 2211–2224. 10.1039/d0fo02848g 33595580

[B35] Wilkinson A. P. Gee J. M. Dupont M. S. Needs P. W. Mellon F. A. Williamson G. (2003). Hydrolysis by lactase phlorizin hydrolase is the first step in the uptake of daidzein glucosides by rat small intestine *in vitro* . Xenobiotica. 33, 255–264. 10.1080/0049825021000058088 12637243

[B36] Wu S. Y. Wang W. J. Dou J. H. Gong L. K. (2021). Research progress on the protective effects of licorice-derived 18β-glycyrrhetinic acid against liver injury. Acta Pharmacol. Sin. 42, 18–26. 10.1038/s41401-020-0383-9 32144337 PMC7921636

[B37] Xu B. L. Li P. Y. Zhang G. J. (2015a). Comparative pharmacokinetics of puerarin, daidzin, baicalin, glycyrrhizic acid, liquiritin, berberine, palmatine and jateorhizine by liquid chromatography-mass spectrometry after oral administration of Gegenqinlian decoction and active components alignment (ACA) to rats. J. Chromatogr. B Anal. Technol. Biomed. Life Sci. 988, 33–44. 10.1016/j.jchromb.2015.01.039 25746576

[B38] Xu J. Lian F. M. Zhao L. H. Zhao Y. F. Chen X. Y. Zhang X. (2015b). Structural modulation of gut microbiota during alleviation of type 2 diabetes with a Chinese herbal formula. Isme J. 9, 552–562. 10.1038/ismej.2014.177 25279787 PMC4331591

[B39] Yan M. Guo L. Yang Y. Zhang B. Hou Z. Gao Y. (2021). Glycyrrhetinic acid protects alpha-naphthylisothiocyanate- induced cholestasis through regulating transporters, inflammation and apoptosis. Front. Pharmacol. 12, 701240. 10.3389/fphar.2021.701240 34630081 PMC8497752

[B40] Yu Z. Yang L. Deng S. Liang M. (2020). Daidzein ameliorates LPS-induced hepatocyte injury by inhibiting inflammation and oxidative stress. Eur. J. Pharmacol. 885, 173399. 10.1016/j.ejphar.2020.173399 32712091

[B41] Zhai L. Huang T. Xiao H. T. Wu P. G. Lin C. Y. Ning Z. W. (2020). Berberine suppresses colonic inflammation in dextran sulfate sodium-induced murine colitis through inhibition of cytosolic phospholipase A2 activity. Front. Pharmacol. 11, 576496. 10.3389/fphar.2020.576496 33658925 PMC7919193

[B42] Zhang C. H. Xiao Q. Sheng J. Q. Liu T. T. Cao Y. Q. Xue Y. N. (2020). Gegen Qinlian Decoction abates nonalcoholic steatohepatitis associated liver injuries via anti-oxidative stress and anti-inflammatory response involved inhibition of toll-like receptor 4 signaling pathways. Biomed. Pharmacother. 126, 110076. ARTN 110076. 10.1016/j.biopha.2020.110076 32169759

[B43] Zhang L. Li C. Lin G. Krajcsi P. Zuo Z. (2011a). Hepatic metabolism and disposition of baicalein via the coupling of conjugation enzymes and transporters-*in vitro* and *in vivo* evidences. AAPS J. 13, 378–389. 10.1208/s12248-011-9277-6 21607811 PMC3160155

[B44] Zhang L. Lin G. Chang Q. Zuo Z. (2005). Role of intestinal first-pass metabolism of baicalein in its absorption process. Pharm. Res. 22, 1050–1058. 10.1007/s11095-005-5303-7 16028005

[B45] Zhang L. Lin G. Zuo Z. (2007). Involvement of UDP-glucuronosyltransferases in the extensive liver and intestinal first-pass metabolism of flavonoid baicalein. Pharm. Res. 24, 81–89. 10.1007/s11095-006-9126-y 17109214

[B46] Zhang Q.-Y. Xu L.-H. Li B.-T. Lu H. Tang X.-L. Xu G.-L. (2011b). Classified and integrated pharmacokinetic study of multipe effective components contained in Gegen-Qinlian decoction. Chin. J. Clin. Pharmacol. Ther. 16, 51–56.

[B47] Zhang Y. F. Yuan J. Wang Y. Wang Y. An R. Wang X. H. (2014). LC-MS/MS determination and pharmacokinetics study of puerarin and daidzein in rat plasma after oral administration of Gegenqinlian decoction and Radix Puerariae extract. Pharmacogn. Mag. 10, 241–248. 10.4103/0973-1296.137363 25210310 PMC4159916

[B48] Zhang Y. F. Yuan J. Zhang Y. Z. Chen Y. Cao J. X. An R. (2015). LC-MS/MS analysis of Gegen Qinlian Decoction and its pharmacokinetics after oral administration to rats. Biomed. Chromatogr. 29, 485–495. 10.1002/bmc.3300 25132315

[B49] Zhao J. Zhao Q. Lu J. Z. Ye D. Mu S. Yang X. D. (2021a). Natural nano-drug delivery system in Coptidis rhizoma extract with modified berberine hydrochloride pharmacokinetics. Int. J. Nanomedicine 16, 6297–6311. 10.2147/IJN.S323685 34552326 PMC8451076

[B50] Zhao J. Zhou T. Lu J. Z. Ye D. Mu S. Tian X. H. (2021b). Intra-herb interactions: Primary metabolites in Coptidis rhizoma extract improved the pharmacokinetics of oral berberine hydrochloride in mice. Front. Pharmacol. 12, 675368. ARTN 675368. 10.3389/fphar.2021.675368 34163360 PMC8215677

[B51] Zhao Y. X. Luan H. F. Gao H. Wu X. J. Zhang Y. B. Li R. Y. (2020). Gegen Qinlian decoction maintains colonic mucosal homeostasis in acute/chronic ulcerative colitis via bidirectionally modulating dysregulated Notch signaling. Phytomedicine 68, 153182. ARTN 153182. 10.1016/j.phymed.2020.153182 32065953

[B52] Zhu H. Qian Z. Li H. Guo L. Pan L. Zhang Q. (2012). Integrated pharmacokinetics of major bioactive components in MCAO rats after oral administration of Huang-Lian-Jie-Du-Tang. J. Ethnopharmacol. 141, 158–169. 10.1016/j.jep.2012.02.014 22387241

