# Supplementary material for: Tissue distribution and integrated pharmacokinetic properties of major effective constituents of oral Gegen-Qinlian decoction in mice
Source: Front Pharmacol. 2022 Oct 11;13:996143. doi: 10.3389/fphar.2022.996143 (PMC9592910; doi:10.3389/fphar.2022.996143)
Supplement: Supplementary file 1 [file DataSheet1.docx]

***Supplementary Materials***

**1 Methodological validation for plasma samples**

**1.1 Selectivity**

The chromatograms of blank plasma, blank plasma spiked with the constituents of GQD at the concentration of their lower limit of quantification (LLOQ), and plasma samples of the mice obtained 1 h after oral administration of GQD were compared to assess the selectivity of the method for each constituent. If the peak area in blank plasma was less than 20% of the peak area of the constituent at the concentration of LLOQ, the selectivity for the constituent was considered qualified. Furthermore, the peak area of the ISs in blank plasma should be less than 5% of the IS peak area in blank plasma spiked with working solution of the ISs. In addition, at least five of the six blank plasma samples from different mice should meet the above criteria.

**1.2 Calibration curves and LLOQs**

The calibration plasma samples were prepared by adding the working solutions to blank plasmas at eight concentrations: 2.5-320.0 ng/mL for puerarin, 2.0-256.0 ng/mL for daidzein, 1.6-200.0 ng/mL for daidzin, 5.0-640.0 ng/mL for baicalin and wogonoside, 3.9-500.0 ng/mL for baicalein, 1.0-128.0 ng/mL for wogonin, berberine hydrochloride, epiberberine and demethyleneberberine, 0.3-32.0 ng/mL for palmatine, 0.5-64.0 ng/mL for coptisine, magnoflorine and jatrorrhizine, 0.8-100.0 ng/mL for berberrubine, 3.1-400.0 ng/mL for liquiritin and liquiritigenin, 30.0-3840.0 ng/mL for glycyrrhizic acid, and 25.0-3200.0 ng/mL for glycyrrhetinic acid, respectively.

The calibration curve of each constituent was established by measuring the peak area ratio of the constituent to corresponding IS with the weighted least-squares linear regression method (1/χ^2^). For each concentration, totally six samples were analyzed in 3 batches, i.e., two samples in each batches. The regression coefficient (r) of each equation should be larger than 0.9950. Additionally, at least 75% of the back-calculated standard concentrations should be within ± 15%, except that of LLOQ, which should be within ± 20%.

The LLOQ of each constituent was the lowest concentration in its calibration curve. The LLOQ should have a higher than 10: 1 signal-to-noise ratio, and could be determined with an accuracy of 80%-120% and a precision of less than 20%. In addition, the LLOQ should be lower than the concentration of the constituent in plasma samples obtained from mice at five T_1/2_ after oral administration of GQD. Alternatively, the LLOQ should be lower than 1/10-1/20 of the peak plasma concentration (C_max_) of the constituent.

**1.3 Precision and accuracy**

The precision and accuracy of the method for each constituent were evaluated in QC samples, which have six replicates at four concentrations. That is, the concentration at LLOQ for each constituent, and additional three concentrations as follow: 7.0, 70.0, 256.0 ng/mL for puerarin, 5.0, 50.0, 200.0 ng/mL for daidzein, 4.0, 40.0, 160.0 ng/mL for daidzin, 15.0, 150.0, 510.0 ng/mL for baicalin and wogonoside, 10.0, 100.0, 400.0 ng/mL for baicalein, 2.0, 20.0, 100.0 ng/mL for wogonin, berberine hydrochloride, epiberberine and demethyleneberberine, 0.5, 5.0, 25.0 ng/mL for palmatine, 1.0, 10.0, 50.0 ng/mL for coptisine, magnoflorine and jatrorrhizine, 2.0, 20.0, 80.0 ng/mL for berberrubine, 9.0, 90.0, 320.0 for liquiritin and liquiritigenin, 80.0, 800.0, 3000.0 ng/mL for glycyrrhizic acid, and 70.0, 700.0, 2560.0 ng/mL for glycyrrhetinic acid, respectively.

The intraday precision and accuracy were evaluated within a day, while the interday precision and accuracy were determined in three separate days with three analytical lots. The actual concentrations of the samples were calculated according to the calibration curves. The accuracy was evaluated by calculating the ratio of the measured concentration to the nominal concentration (relative error, RE), which should be within ± 20% for the concentration of LLOQ and within ± 15% for other concentrations. The precision were assessed by the relative standard deviation (RSD), which should be less than 20% for the concentration of LLOQ and less than 15% for other concentrations.

**1.4 Carry-over**

The blank plasma samples were spiked with the working solutions of the ISs and the constituents of GQD at the concentration of their upper limit of quantification (ULOQ). Then the samples were injected in the LC-MS/MS system. After that, the double blank samples without GQD constituents and the ISs were injected for analysis. In the double blank samples, the average peak area of each GQD constituent should be less than 20% of their peak area at the concentration of LLOQ, while the average peak area of the ISs should be less than 5% of their average responses in the QCs samples.

**1.5 Recovery and matrix effects**

The recovery and matrix effects of each GQD constituent were studied in QC samples, which have three concentrations with six replicates. The QC samples of GQD constituents prepared according to “2.3.2” were named as QC-As. The QC samples prepared by spiking the blank plasma matrix with corresponding concentrations of GQD constituents with QC-As were named as QC-Bs. The recovery of GQD constituents was then determined by comparing the peak areas of GQD constituents in QC-As with corresponding QC-Bs. The reproducibility of the extraction procedure was evaluated according to the RSD of the recovery, which should be less than 15%. When methanol was used to prepare the QC samples instead of blank plasma, the samples were named as QC-Cs. The matrix effects of GQD constituents were then determined by comparing their peak areas in QC-Bs to those in QC-Cs at corresponding concentrations. The reproducibility of the matrix effects were determined according to RSD and should be less than 15%.

**1.6 Stability**

The stability of GQD constituents was investigated by analyzing the repeatability of plasma samples at three concentration levels under three different conditions as follows: short-term stability (room temperature for 2 h), post-preparative stability (4°C for 24 h), and long-term stability (−80°C for 7 days). The concentration of each GQD constituent was measured by the calibration curve that was calculated based on freshly prepared calibration samples. A GQD constituent was considered stable if the accuracy of at least 2/3 of the samples was within ± 15%, and the RSD was less than 15%.

**1.7 Dilution linearity**

Dilution linearity of the method was investigated by spiking in blank plasma with GQD constituents at the concentration three times of their ULOQs. Then the QC samples were diluted ten times with blank plasma. The dilution linearity was determined in five replicates. The accuracies should be within ± 15% of nominal concentrations, and the precisions should be less than 15%.

**2 Partial methodological validation for the liver homogenate samples**

Partial methodological validation for the liver homogenate samples was performed in terms of specificity, standard curve, linear range, LLOQ, intraday precision and accuracy, carry over, recovery and matrix effect, and stability. Regarding linear range, baicalein is within 5.0-640.0 ng/mL, glycyrrhizic acid is within 25.0-3200.0 ng/mL, the linear ranges of other constituents were the same as those in the plasma samples. For QC samples, the low, medium and high concentrations of baicalein were 15.0, 150.0, and 510.0 ng/mL, respectively; the low, medium and high concentrations of glycyrrhizic acid were 70.0, 700.0 and 2560.0 ng/mL, respectively; while the QC samples of other constituents were the same as those in the plasma samples. Only intraday precision and accuracy were examined at low, medium and high concentrations of QC samples. Matrix effects were examined at low and high QC concentrations of QC samples. Stability was examined at low and high QC concentrations of QC samples. The rest of the methodological validations were the same as those in plasma samples.

**3 Supplementary data**

**3.1 Supplementary tables**

Table S1. Instrument methods of the liquid chromatography tandem mass spectrometry for 19 target analysts and the internal standards (ISs).

| Constituents | m/z | Q1  (Da) | Q3  (Da) | Declustering  potential (V) | Collision  energy  (eV) | Collision cell  exit potential  (V) |
| --- | --- | --- | --- | --- | --- | --- |
| Puerarin | [M+H]^+^ | 417.1 | 297.1 | 76.9 | 37.4 | 13.1 |
| Daidzein | [M+H]^+^ | 255.3 | 199.3 | 162.7 | 34.8 | 23.2 |
| Daidzin | [M+H]^+^ | 417.4 | 255.1 | 86.1 | 20.4 | 13.0 |
| Baicalin | [M+H]^+^ | 447.4 | 271.1 | 46.9 | 26.0 | 17.8 |
| Wogonoside | [M+H]^+^ | 461.4 | 285.2 | 169.7 | 23.8 | 23.7 |
| Baicalein | [M+H]^+^ | 271.1 | 123.1 | 112.2 | 40.2 | 19.1 |
| Wogonin | [M+H]^+^ | 285.2 | 270.2 | 89.9 | 25.0 | 12.9 |
| Berberine | [M]^+^ | 336.1 | 321.3 | 69.7 | 22.0 | 16.6 |
| Palmatine | [M]^+^ | 352.3 | 337.2 | 50.8 | 30.0 | 21.4 |
| Coptisine | [M]^+^ | 320.7 | 293.1 | 68.7 | 40.0 | 27.0 |
| Epiberberine | [M]^+^ | 336.2 | 321.0 | 55.9 | 18.0 | 15.2 |
| Magnoflorine | [M]^+^ | 342.1 | 297.2 | 45.1 | 25.5 | 19.7 |
| Demethylene-  berberine | [M]^+^ | 324.9 | 309.1 | 69.2 | 39.2 | 28.2 |
| Berberrubine | [M]^+^ | 322.2 | 307.2 | 85.2 | 25.0 | 21.3 |
| Jatrorrhizine | [M]^+^ | 338.1 | 323.3 | 53.3 | 41.5 | 16.5 |
| Liquiritin | [M-H]^-^ | 417.2 | 255.0 | -103.1 | -27.9 | -28.2 |
| Liquiritigenin | [M-H]^-^ | 255.2 | 119.0 | -90.1 | -28.3 | -12.4 |
| Glycyrrhizic  acid | [M-H]^-^ | 821.4 | 351.1 | -220.2 | -55.4 | -35.6 |
| Glycyrrhetinic  acid | [M-H]^-^ | 469.3 | 355.0 | -212.9 | -63.4 | -43.2 |
| Naringin (IS1) | [M+Na]^+^ | 603.2 | 457.1 | 130.8 | 33.9 | 21.1 |
| Mycophenolic  acid (IS2) | [M-H]^-^ | 319.0 | 191.0 | -82.5 | -30.2 | -24.1 |

Table S2. Instrument conditions of the liquid chromatography tandem mass spectrometry for 17 target analysts and the internal standards (ISs).

| Constituents | m/z | Q1  (Da) | Q3  (Da) | Declustering  potential (V) | Collision  energy  (eV) | Collision cell  exit potential  (V) |
| --- | --- | --- | --- | --- | --- | --- |
| Puerarin | [M+H]^+^ | 417.2 | 297.0 | 62.1 | 36.7 | 18.8 |
| Daidzein | [M+H]^+^ | 255.1 | 199.1 | 139.1 | 32.67 | 23.89 |
| Daidzin | [M+H]^+^ | 417.3 | 255.1 | 103.1 | 29.6 | 20.9 |
| Baicalin | [M+H]^+^ | 447.2 | 271.1 | 40.9 | 23.0 | 13.5 |
| Wogonoside | [M+H]^+^ | 461.4 | 285.2 | 169.7 | 23.8 | 23.7 |
| Baicalein | [M+H]^+^ | 271.2 | 123.0 | 162.7 | 41.2 | 15.7 |
| Wogonin | [M+H]^+^ | 285.1 | 270.2 | 109.2 | 33.4 | 15.6 |
| Berberine | [M]^+^ | 336.1 | 321.2 | 29.1 | 41.3 | 15.5 |
| Palmatine | [M]^+^ | 353.0 | 337.2 | 51.5 | 39.2 | 20.4 |
| Coptisine | [M]^+^ | 320.7 | 293.2 | 78.4 | 37.3 | 14.1 |
| Epiberberine | [M]^+^ | 336.2 | 320.2 | 39.6 | 40.8 | 14.8 |
| Magnoflorine | [M]^+^ | 342.1 | 297.2 | 101.9 | 28.3 | 23.9 |
| Demethylene-  berberine | [M]^+^ | 324.1 | 308.2 | 97.9 | 42.3 | 17.8 |
| Jatrorrhizine | [M]^+^ | 338.8 | 323.2 | 28.3 | 38.2 | 18.3 |
| Isoliquiritin | [M-H]^-^ | 417.2 | 255.1 | -181.9 | -25.9 | -10.9 |
| Liquiritin | [M-H]^-^ | 417.1 | 255.0 | -109.06 | -28.6 | -11.1 |
| Glycyrrhizic  acid | [M-H]^-^ | 821.1 | 351.1 | -58.3 | -53.0 | -14.7 |
| Naringin (IS1) | [M+Na]^+^ | 603.2 | 457.1 | 130.8 | 33.9 | 21.1 |
| Mycophenolic  acid (IS2) | [M-H]^-^ | 319.0 | 191.0 | -82.5 | -30.2 | -24.1 |

Table S3. Intraday and interday precision and accuracy of the constituents of *Gegen-Qinlian* decoction in quality control plasma samples (mean ± SD, n = 6 for intraday and n = 6×3 for interday).

| Constituents | Nominal  concentrations  (ng/mL) | Intraday | | | | | Interday | | | | |
| --- | --- | --- | --- | --- | --- | --- | --- | --- | --- | --- | --- |
|  |  | Determined  concentrations (ng/mL) | | | RSD (%) | RE (%) | Determined  concentrations (ng/mL) | | | RSD (%) | RE (%) |
| Puerarin | 2.5 | 2.4 | ± | 0.3 | 11.4 | 96.3 | 2.4 | ± | 0.1 | 2.1 | 96.4 |
|  | 7.0 | 6.7 | ± | 0.6 | 9.4 | 95.1 | 6.8 | ± | 0.2 | 3.6 | 96.6 |
|  | 70.0 | 66.4 | ± | 2.1 | 3.2 | 94.8 | 68.3 | ± | 2.4 | 3.5 | 97.5 |
|  | 256.0 | 245.8 | ± | 5.3 | 2.2 | 96.0 | 248.2 | ± | 5.5 | 2.2 | 96.9 |
| Daidzein | 2.0 | 2.2 | ± | 0.2 | 9.8 | 112.4 | 2.1 | ± | 0.1 | 5.2 | 106.3 |
|  | 5.0 | 5.1 | ± | 0.3 | 5.4 | 102.0 | 5.0 | ± | 0.6 | 11.9 | 100.8 |
|  | 50.0 | 49.0 | ± | 2.6 | 5.2 | 98.0 | 47.8 | ± | 1.6 | 3.4 | 95.5 |
|  | 200.0 | 197.7 | ± | 5.5 | 2.8 | 98.8 | 189.4 | ± | 7.6 | 4.0 | 94.7 |
| Daidzin | 1.6 | 1.9 | ± | 0.3 | 18.6 | 118.6 | 1.8 | ± | 0.1 | 5.2 | 114.4 |
|  | 4.0 | 3.6 | ± | 0.3 | 9.1 | 89.2 | 3.8 | ± | 0.2 | 5.1 | 94.4 |
|  | 40.0 | 38.8 | ± | 3.1 | 7.9 | 97.1 | 38.3 | ± | 1.6 | 4.1 | 95.8 |
|  | 160.0 | 156.8 | ± | 2.6 | 1.7 | 98.0 | 157.6 | ± | 4.3 | 2.7 | 98.5 |
| Baicalin | 5.0 | 4.8 | ± | 0.4 | 8.2 | 96.2 | 5.2 | ± | 0.4 | 7.7 | 104.5 |
|  | 15.0 | 13.5 | ± | 0.5 | 3.7 | 90.2 | 14.4 | ± | 0.8 | 5.8 | 96.3 |
|  | 150.0 | 147.2 | ± | 11.4 | 7.8 | 98.1 | 145.5 | ± | 5.4 | 3.7 | 97.0 |
|  | 510.0 | 509.7 | ± | 5.1 | 1.0 | 99.9 | 506.2 | ± | 4.9 | 1.0 | 99.3 |
| Wogonoside | 5.0 | 4.3 | ± | 0.7 | 15.1 | 86.8 | 4.7 | ± | 0.3 | 7.3 | 93.0 |
|  | 15.0 | 13.0 | ± | 1.1 | 8.5 | 86.6 | 13.5 | ± | 0.5 | 3.6 | 90.1 |
|  | 150.0 | 136.8 | ± | 6.3 | 4.6 | 91.2 | 143.6 | ± | 6.5 | 4.5 | 95.7 |
|  | 510.0 | 495.5 | ± | 18.0 | 3.6 | 97.2 | 500.7 | ± | 5.3 | 1.0 | 98.2 |
| Baicalein | 3.9 | 3.3 | ± | 0.4 | 12.9 | 84.3 | 3.7 | ± | 0.3 | 9.4 | 94.0 |
|  | 10.0 | 9.8 | ± | 0.9 | 9.1 | 97.8 | 11.0 | ± | 1.2 | 11.2 | 109.8 |
|  | 100.0 | 107.8 | ± | 5.4 | 5.0 | 107.8 | 107.9 | ± | 10.2 | 9.4 | 107.9 |
|  | 400.0 | 430.2 | ± | 18.3 | 4.3 | 107.5 | 419.8 | ± | 20.5 | 4.9 | 104.9 |
| Wogonin | 1.0 | 0.9 | ± | 0.1 | 8.9 | 92.6 | 0.9 | ± | 0.0 | 1.4 | 92.9 |
|  | 2.0 | 1.8 | ± | 0.2 | 8.6 | 89.8 | 1.8 | ± | 0.0 | 2.0 | 91.7 |
|  | 20.0 | 19.0 | ± | 1.4 | 7.5 | 94.8 | 19.6 | ± | 0.8 | 4.2 | 97.9 |
|  | 100.0 | 101.2 | ± | 2.1 | 2.1 | 101.2 | 106.2 | ± | 4.8 | 4.5 | 106.2 |
| Berberine | 1.0 | 1.1 | ± | 0.2 | 15.6 | 106.2 | 1.0 | ± | 0.0 | 1.5 | 104.9 |
|  | 2.0 | 2.0 | ± | 0.3 | 12.5 | 101.1 | 2.0 | ± | 0.0 | 2.4 | 101.5 |
|  | 20.0 | 19.0 | ± | 1.4 | 7.5 | 94.8 | 19.6 | ± | 0.8 | 4.2 | 97.9 |
|  | 100.0 | 104.3 | ± | 9.5 | 9.1 | 104.3 | 100.2 | ± | 4.0 | 4.0 | 100.2 |
| Palmatine | 0.3 | 0.2 | ± | 0.0 | 17.1 | 98.3 | 0.2 | ± | 0.0 | 3.7 | 99.6 |
|  | 0.5 | 0.5 | ± | 0.1 | 11.9 | 100.1 | 0.5 | ± | 0.0 | 2.9 | 102.5 |
|  | 5.0 | 4.9 | ± | 0.2 | 4.4 | 98.6 | 5.0 | ± | 0.2 | 4.8 | 100.6 |
|  | 25.0 | 23.3 | ± | 0.8 | 3.5 | 93.3 | 23.4 | ± | 0.2 | 0.8 | 93.7 |
| Coptisine | 0.5 | 0.5 | ± | 0.1 | 11.3 | 106.9 | 0.5 | ± | 0.1 | 10.6 | 103.5 |
|  | 1.0 | 0.9 | ± | 0.1 | 7.6 | 85.5 | 1.0 | ± | 0.1 | 12.0 | 95.0 |
|  | 10.0 | 9.5 | ± | 0.4 | 3.8 | 95.0 | 10.0 | ± | 0.9 | 8.8 | 99.7 |
|  | 50.0 | 48.1 | ± | 1.3 | 2.8 | 96.2 | 48.5 | ± | 1.0 | 2.1 | 97.0 |
| Epiberberine | 1.0 | 0.9 | ± | 0.1 | 16.0 | 92.8 | 1.0 | ± | 0.1 | 10.9 | 102.0 |
|  | 2.0 | 1.9 | ± | 0.2 | 10.8 | 93.1 | 1.9 | ± | 0.1 | 3.1 | 96.5 |
|  | 20.0 | 17.5 | ± | 1.3 | 7.4 | 87.7 | 18.7 | ± | 1.6 | 8.3 | 93.7 |
|  | 100.0 | 103.7 | ± | 8.3 | 8.0 | 103.7 | 101.3 | ± | 7.5 | 7.4 | 101.3 |
| Magnoflorine | 0.5 | 0.5 | ± | 0.0 | 9.2 | 92.9 | 0.5 | ± | 0.0 | 6.0 | 98.8 |
|  | 1.0 | 0.9 | ± | 0.1 | 10.3 | 94.5 | 1.0 | ± | 0.1 | 6.0 | 97.5 |
|  | 10.0 | 8.8 | ± | 0.5 | 5.2 | 88.2 | 9.2 | ± | 0.7 | 7.7 | 92.3 |
|  | 50.0 | 46.5 | ± | 1.3 | 2.8 | 93.0 | 47.3 | ± | 1.6 | 3.5 | 94.6 |
| Demethyleneberberine | 1.0 | 0.9 | ± | 0.1 | 14.9 | 92.0 | 1.1 | ± | 0.1 | 12.7 | 107.8 |
|  | 2.0 | 1.8 | ± | 0.1 | 3.7 | 91.8 | 2.0 | ± | 0.2 | 10.5 | 100.2 |
|  | 20.0 | 20.5 | ± | 1.4 | 6.7 | 102.6 | 20.8 | ± | 1.2 | 5.9 | 104.2 |
|  | 100.0 | 98.2 | ± | 3.7 | 3.8 | 98.2 | 96.5 | ± | 5.0 | 5.2 | 96.5 |
| Berberrubine | 0.8 | 0.7 | ± | 0.1 | 19.1 | 95.0 | 0.7 | ± | 0.0 | 4.0 | 91.2 |
|  | 2.0 | 2.3 | ± | 0.1 | 5.1 | 113.2 | 2.2 | ± | 0.2 | 7.9 | 108.4 |
|  | 20.0 | 22.6 | ± | 0.9 | 3.9 | 113.1 | 20.6 | ± | 2.3 | 11.3 | 103.1 |
|  | 80.0 | 91.4 | ± | 2.5 | 2.7 | 114.3 | 83.4 | ± | 7.4 | 8.9 | 104.3 |
| Jatrorrhizine | 0.5 | 0.5 | ± | 0.0 | 9.0 | 97.6 | 0.5 | ± | 0.0 | 2.4 | 100.3 |
|  | 1.0 | 1.0 | ± | 0.1 | 11.2 | 96.3 | 1.0 | ± | 0.0 | 4.2 | 98.4 |
|  | 10.0 | 9.6 | ± | 0.6 | 6.3 | 95.5 | 9.9 | ± | 0.7 | 7.4 | 99.2 |
|  | 50.0 | 47.6 | ± | 1.7 | 3.5 | 95.2 | 47.4 | ± | 2.3 | 4.8 | 94.9 |
| Liquiritin | 3.1 | 3.6 | ± | 0.7 | 19.1 | 114.5 | 3.1 | ± | 0.5 | 14.8 | 98.1 |
|  | 9.0 | 8.7 | ± | 0.2 | 2.3 | 97.2 | 8.4 | ± | 0.3 | 4.0 | 92.9 |
|  | 90.0 | 85.5 | ± | 7.3 | 8.5 | 95.0 | 87.0 | ± | 3.2 | 3.6 | 96.7 |
|  | 320.0 | 312.7 | ± | 7.6 | 2.4 | 97.7 | 301.3 | ± | 10.2 | 3.4 | 94.2 |
| Liquiritigenin | 3.1 | 3.2 | ± | 0.3 | 8.3 | 101.3 | 3.1 | ± | 0.1 | 4.4 | 100.6 |
|  | 9.0 | 9.4 | ± | 0.9 | 9.7 | 105.0 | 9.2 | ± | 0.3 | 3.4 | 101.8 |
|  | 90.0 | 84.0 | ± | 6.0 | 7.1 | 93.3 | 86.1 | ± | 3.1 | 3.6 | 95.7 |
|  | 320.0 | 310.8 | ± | 4.6 | 1.5 | 97.1 | 308.9 | ± | 6.1 | 2.0 | 96.5 |
| Glycyrrhizic acid | 30.0 | 32.5 | ± | 2.0 | 6.3 | 108.2 | 31.6 | ± | 1.1 | 3.4 | 105.3 |
|  | 80.0 | 82.8 | ± | 5.1 | 6.1 | 103.4 | 82.6 | ± | 1.2 | 1.5 | 103.3 |
|  | 800.0 | 805.2 | ± | 84.6 | 10.5 | 100.6 | 790.9 | ± | 13.3 | 1.7 | 98.9 |
|  | 3000.0 | 3270.0 | ± | 287.9 | 8.8 | 109.0 | 3007.2 | ± | 230.9 | 7.7 | 100.2 |
| Glycyrrhetinic acid | 25.0 | 22.8 | ± | 1.0 | 4.2 | 91.1 | 25.0 | ± | 2.0 | 7.8 | 100.1 |
|  | 70.0 | 62.0 | ± | 6.7 | 10.8 | 88.5 | 66.8 | ± | 4.3 | 6.4 | 95.4 |
|  | 700.0 | 654.2 | ± | 39.9 | 6.1 | 93.5 | 700.6 | ± | 45.2 | 6.5 | 100.1 |
|  | 2560.0 | 2303.3 | ± | 101.9 | 4.4 | 90.0 | 2425.6 | ± | 108.4 | 4.5 | 94.7 |

Table S4. Carry-over of the constituents of *Gegen*-*Qinlian* decoction and internal standard (ISs) in quality control plasma samples.

| Constituents | Peak area (counts) | | Carry-over (%) |
| --- | --- | --- | --- |
| Puerarin | LLOQ | 3.01E+04 | 1.7 |
|  | DB | 5.17E+02 |  |
| Daidzein | LLOQ | 3.07E+04 | 5.4 |
|  | DB | 1.66E+03 |  |
| Daidzin | LLOQ | 1.54E+04 | 4.6 |
|  | DB | 7.16E+02 |  |
| Baicalin | LLOQ | 1.96E+04 | 8.6 |
|  | DB | 1.68E+03 |  |
| Wogonoside | LLOQ | 1.24E+04 | 2.1 |
|  | DB | 2.58E+02 |  |
| Baicalein | LLOQ | 1.63E+04 | 5.2 |
|  | DB | 8.54E+02 |  |
| Wogonin | LLOQ | 2.96E+04 | 6.3 |
|  | DB | 1.86E+03 |  |
| Berberine | LLOQ | 6.06E+05 | 0.6 |
|  | DB | 3.68E+03 |  |
| Palmatine | LLOQ | 6.25E+05 | 0.2 |
|  | DB | 1.39E+03 |  |
| Coptisine | LLOQ | 4.52E+04 | 0.2 |
|  | DB | 1.04E+02 |  |
| Epiberberine | LLOQ | 2.07E+05 | 0.5 |
|  | DB | 1.00E+03 |  |
| Magnoflorine | LLOQ | 7.38E+04 | 1.4 |
|  | DB | 1.06E+03 |  |
| Demethyleneberberine | LLOQ | 1.89E+04 | 1.4 |
|  | DB | 2.64E+02 |  |
| Berberrubine | LLOQ | 6.29E+03 | 12.1 |
|  | DB | 7.61E+02 |  |
| Jatrorrhizine | LLOQ | 7.67E+04 | 0.3 |
|  | DB | 2.42E+02 |  |
| Liquiritin | LLOQ | 1.46E+04 | 0.7 |
|  | DB | 9.85E+01 |  |
| Liquiritigenin | LLOQ | 3.99E+04 | 2.8 |
|  | DB | 1.12E+03 |  |
| Glycyrrhizic acid | LLOQ | 5.15E+02 | 3.6 |
|  | DB | 1.85E+01 |  |
| Glycyrrhetinic acid | LLOQ | 3.31E+03 | 3.4 |
|  | DB | 1.12E+02 |  |
| Naringin (IS1) | LLOQ | 3.88E+05 | 0.1 |
|  | DB | 2.02E+02 |  |
| Mycophenolic acid (IS2) | LLOQ | 2.96E+04 | 2.7 |
|  | DB | 7.85E+02 |  |

LLOQ，plasma matrix with lower limit of quantification；DB，double blank plasma matrix

Table S5. Recovery and matrix effects of the constituents of *Gegen*-*Qinlian* decoction and internal standards (ISs) in quality control plasma samples (mean ± SD, n = 6).

| Constituents | Nominal  concentrations  (ng/mL) | Recovery (%) | | | | Matrix effect (%) | | | |
| --- | --- | --- | --- | --- | --- | --- | --- | --- | --- |
|  |  | Mean | ± | SD | RSD (%) | Mean | ± | SD | RSD (%) |
| Puerarin | 7.0 | 107.3 | ± | 4.4 | 4.1 | 93.2 | ± | 2.5 | 2.7 |
|  | 70.0 | 100.9 | ± | 5.9 | 5.8 | 101.1 | ± | 3.4 | 3.3 |
|  | 256.0 | 97.9 | ± | 4.9 | 5.0 | 105.9 | ± | 4.3 | 4.0 |
| Daidzein | 5.0 | 93.1 | ± | 3.6 | 3.8 | 102.9 | ± | 11.7 | 11.4 |
|  | 50.0 | 112.0 | ± | 2.5 | 2.2 | 109.3 | ± | 2.6 | 2.3 |
|  | 200.0 | 108.3 | ± | 4.2 | 3.9 | 97.2 | ± | 6.6 | 6.8 |
| Daidzin | 4.0 | 102.9 | ± | 5.1 | 4.9 | 98.7 | ± | 5.6 | 5.7 |
|  | 40.0 | 109.4 | ± | 5.4 | 4.9 | 104.3 | ± | 7.9 | 7.5 |
|  | 160.0 | 100.2 | ± | 2.0 | 2.0 | 113.6 | ± | 3.6 | 3.1 |
| Baicalin | 15.0 | 96.6 | ± | 5.9 | 6.1 | 101.7 | ± | 3.2 | 3.1 |
|  | 150.0 | 94.0 | ± | 5.9 | 6.3 | 98.5 | ± | 3.0 | 3.0 |
|  | 510.0 | 99.5 | ± | 2.5 | 2.5 | 104.2 | ± | 3.9 | 3.7 |
| Wogonoside | 15.0 | 102.9 | ± | 7.1 | 6.9 | 100.1 | ± | 5.1 | 5.1 |
|  | 150.0 | 94.8 | ± | 4.3 | 4.6 | 109.7 | ± | 6.6 | 6.0 |
|  | 510.0 | 95.5 | ± | 2.9 | 3.0 | 101.5 | ± | 1.9 | 1.9 |
| Baicalein | 10.0 | 98.5 | ± | 13.5 | 13.7 | 102.0 | ± | 7.3 | 7.2 |
|  | 100.0 | 94.3 | ± | 4.2 | 4.5 | 114.6 | ± | 3.1 | 2.7 |
|  | 400.0 | 96.8 | ± | 3.7 | 3.8 | 113.8 | ± | 7.7 | 6.8 |
| Wogonin | 2.0 | 105.1 | ± | 2.8 | 2.6 | 96.9 | ± | 3.4 | 3.5 |
|  | 20.0 | 101.6 | ± | 6.9 | 6.8 | 100.4 | ± | 3.8 | 3.8 |
|  | 100.0 | 89.6 | ± | 5.2 | 5.8 | 113.9 | ± | 6.8 | 6.0 |
| Berberine | 2.0 | 103.7 | ± | 10.3 | 10.0 | 100.0 | ± | 2.4 | 2.4 |
|  | 20.0 | 102.5 | ± | 11.4 | 11.2 | 102.5 | ± | 6.6 | 6.5 |
|  | 100.0 | 96.5 | ± | 3.4 | 3.5 | 101.0 | ± | 0.8 | 0.8 |
| Palmatine | 0.5 | 103.6 | ± | 6.1 | 5.9 | 100.2 | ± | 3.0 | 3.0 |
|  | 5.0 | 103.7 | ± | 13.2 | 12.8 | 97.4 | ± | 5.2 | 5.4 |
|  | 25.0 | 92.1 | ± | 7.3 | 8.0 | 114.4 | ± | 1.8 | 1.6 |
| Coptisine | 1.0 | 99.3 | ± | 2.6 | 2.6 | 99.6 | ± | 1.7 | 1.7 |
|  | 10.0 | 99.2 | ± | 12.0 | 12.0 | 97.1 | ± | 2.5 | 2.6 |
|  | 50.0 | 91.0 | ± | 2.5 | 2.7 | 100.7 | ± | 2.9 | 2.9 |
| Epiberberine | 2.0 | 99.0 | ± | 9.6 | 9.7 | 94.0 | ± | 3.4 | 3.6 |
|  | 20.0 | 98.7 | ± | 14.7 | 14.9 | 111.0 | ± | 6.2 | 5.6 |
|  | 100.0 | 92.9 | ± | 6.8 | 7.4 | 110.6 | ± | 6.9 | 6.2 |
| Magnoflorine | 1.0 | 101.5 | ± | 8.7 | 8.6 | 86.8 | ± | 2.9 | 3.3 |
|  | 10.0 | 98.1 | ± | 8.0 | 8.1 | 90.7 | ± | 2.5 | 2.8 |
|  | 50.0 | 90.3 | ± | 2.6 | 2.9 | 103.7 | ± | 1.1 | 1.0 |
| Demethyleneberberine | 2.0 | 95.9 | ± | 4.7 | 4.9 | 91.7 | ± | 6.9 | 7.5 |
|  | 20.0 | 98.7 | ± | 6.7 | 6.8 | 104.9 | ± | 4.2 | 4.0 |
|  | 100.0 | 85.1 | ± | 2.5 | 3.0 | 105.2 | ± | 2.0 | 1.9 |
| Berberrubine | 2.0 | 91.5 | ± | 7.6 | 8.3 | 93.4 | ± | 1.6 | 1.7 |
|  | 20.0 | 92.5 | ± | 5.7 | 6.2 | 107.0 | ± | 2.5 | 2.3 |
|  | 80.0 | 85.9 | ± | 5.4 | 6.2 | 112.8 | ± | 3.3 | 2.9 |
| Jatrorrhizine | 1.0 | 98.8 | ± | 4.8 | 4.8 | 86.5 | ± | 3.2 | 3.7 |
|  | 10.0 | 99.6 | ± | 12.0 | 12.0 | 100.7 | ± | 3.4 | 3.4 |
|  | 50.0 | 85.7 | ± | 5.3 | 6.2 | 110.7 | ± | 5.1 | 4.6 |
| Liquiritin | 9.0 | 112.1 | ± | 7.7 | 6.8 | 88.7 | ± | 3.3 | 3.7 |
|  | 90.0 | 106.7 | ± | 7.5 | 7.0 | 102.0 | ± | 3.2 | 3.1 |
|  | 320.0 | 99.5 | ± | 2.0 | 2.0 | 111.9 | ± | 8.8 | 7.9 |
| Liquiritigenin | 9.0 | 101.2 | ± | 4.0 | 3.9 | 98.8 | ± | 4.9 | 5.0 |
|  | 90.0 | 106.0 | ± | 6.8 | 6.4 | 100.8 | ± | 5.7 | 5.6 |
|  | 320.0 | 100.1 | ± | 6.0 | 6.0 | 108.9 | ± | 2.1 | 1.9 |
| Glycyrrhizic acid | 80.0 | 92.7 | ± | 5.7 | 6.1 | 90.1 | ± | 2.6 | 2.9 |
|  | 800.0 | 98.2 | ± | 4.1 | 4.2 | 91.2 | ± | 3.4 | 3.8 |
|  | 3000.0 | 86.0 | ± | 3.1 | 3.6 | 93.4 | ± | 4.3 | 4.6 |
| Glycyrrhetinic acid | 70.0 | 98.7 | ± | 3.6 | 3.6 | 94.5 | ± | 2.9 | 3.1 |
|  | 700.0 | 97.7 | ± | 3.8 | 3.8 | 97.6 | ± | 1.1 | 1.1 |
|  | 2560.0 | 100.7 | ± | 2.7 | 2.6 | 96.6 | ± | 3.0 | 3.1 |
| Naringin (IS1) | 20000.0 | 99.8 | ± | 1.9 | 1.9 | 97.4 | ± | 1.5 | 1.5 |
|  |  | 99.4 | ± | 1.4 | 1.4 | 102.0 | ± | 1.1 | 1.1 |
|  |  | 100.0 | ± | 2.5 | 2.5 | 101.9 | ± | 2.6 | 2.5 |
| Mycophenolic acid (IS2) | 150.0 | 98.8 | ± | 2.2 | 2.2 | 99.2 | ± | 1.4 | 1.4 |
|  |  | 97.9 | ± | 1.9 | 1.9 | 102.2 | ± | 1.6 | 1.6 |
|  |  | 99.9 | ± | 1.9 | 1.9 | 101.5 | ± | 1.8 | 1.8 |

Table S6. Stability of the constituents of *Gegen*-*Qinlian* decoction in stored quality control plasma samples (mean ± SD, n = 3).

| Constituents | Nominal  concentrations  (ng/mL) | -80°C for 7days | | | | | 4°C for 24 h | | | | | 22°C for 2 h | | | | |
| --- | --- | --- | --- | --- | --- | --- | --- | --- | --- | --- | --- | --- | --- | --- | --- | --- |
|  |  | Determined  concentrations  (ng/mL) | | | RSD  (%) | RE  (%) | Determined  concentrations  (ng/mL) | | | RSD  (%) | RE  (%) | Determined  concentrations  (ng/mL) | | | RSD  (%) | RE  (%) |
| Puerarin | 7.0 | 7.0 | ± | 0.3 | 3.8 | 100.5 | 6.6 | ± | 0.5 | 6.9 | 94.1 | 6.9 | ± | 0.3 | 3.8 | 98.9 |
|  | 70.0 | 67.0 | ± | 3.9 | 5.8 | 95.7 | 66.4 | ± | 6.2 | 9.4 | 94.9 | 66.5 | ± | 0.4 | 0.6 | 95.0 |
|  | 256.0 | 254.0 | ± | 9.6 | 3.8 | 99.2 | 245.3 | ± | 7.4 | 3.0 | 95.8 | 243.0 | ± | 4.6 | 1.9 | 94.9 |
| Daidzein | 5.0 | 4.5 | ± | 0.3 | 5.7 | 90.9 | 4.4 | ± | 0.3 | 7.5 | 88.1 | 5.0 | ± | 0.3 | 5.5 | 99.3 |
|  | 50.0 | 45.6 | ± | 1.9 | 4.2 | 91.3 | 48.0 | ± | 2.3 | 4.8 | 95.9 | 49.6 | ± | 3.3 | 6.6 | 99.2 |
|  | 200.0 | 200.7 | ± | 15.7 | 7.8 | 100.3 | 185.7 | ± | 3.8 | 2.0 | 92.8 | 194.3 | ± | 2.3 | 1.2 | 97.2 |
| Daidzin | 4.0 | 3.8 | ± | 0.1 | 3.7 | 95.3 | 3.9 | ± | 0.2 | 4.4 | 97.0 | 3.7 | ± | 0.3 | 8.6 | 93.5 |
|  | 40.0 | 34.4 | ± | 0.7 | 1.9 | 86.0 | 36.7 | ± | 3.1 | 8.4 | 91.8 | 40.0 | ± | 4.0 | 10.0 | 100.1 |
|  | 160.0 | 155.7 | ± | 10.4 | 6.7 | 97.3 | 154.0 | ± | 7.0 | 4.5 | 96.3 | 158.0 | ± | 2.6 | 1.7 | 98.8 |
| Baicalin | 15.0 | 16.3 | ± | 1.8 | 10.8 | 108.4 | 14.6 | ± | 0.6 | 3.9 | 97.6 | 13.7 | ± | 0.3 | 2.1 | 91.1 |
|  | 150.0 | 145.3 | ± | 4.0 | 2.8 | 96.9 | 145.7 | ± | 8.1 | 5.6 | 97.1 | 153.7 | ± | 13.2 | 8.6 | 102.4 |
|  | 510.0 | 506.0 | ± | 22.5 | 4.4 | 99.2 | 499.7 | ± | 5.1 | 1.0 | 98.0 | 509.7 | ± | 2.3 | 0.5 | 99.9 |
| Wogonoside | 15.0 | 13.9 | ± | 0.8 | 5.5 | 92.9 | 13.2 | ± | 1.0 | 7.5 | 88.0 | 13.2 | ± | 0.9 | 6.8 | 88.0 |
|  | 150.0 | 151.3 | ± | 1.5 | 1.0 | 100.9 | 145.0 | ± | 10.0 | 6.9 | 96.7 | 137.3 | ± | 7.2 | 5.3 | 91.6 |
|  | 510.0 | 513.3 | ± | 25.9 | 5.0 | 100.7 | 503.0 | ± | 11.4 | 2.3 | 98.6 | 504.0 | ± | 15.7 | 3.1 | 98.8 |
| Baicalein | 10.0 | 11.3 | ± | 0.9 | 7.5 | 112.7 | 11.4 | ± | 0.4 | 3.1 | 113.5 | 10.7 | ± | 0.8 | 7.3 | 106.5 |
|  | 100.0 | 93.7 | ± | 3.4 | 3.6 | 93.7 | 98.6 | ± | 4.7 | 4.8 | 98.6 | 108.7 | ± | 7.6 | 7.0 | 108.7 |
|  | 400.0 | 420.0 | ± | 19.2 | 4.6 | 105.0 | 396.7 | ± | 8.7 | 2.2 | 99.2 | 426.7 | ± | 19.5 | 4.6 | 106.7 |
| Wogonin | 2.0 | 1.8 | ± | 0.1 | 3.2 | 92.2 | 1.9 | ± | 0.1 | 6.8 | 92.7 | 1.7 | ± | 0.2 | 8.7 | 86.5 |
|  | 20.0 | 19.4 | ± | 1.5 | 7.5 | 97.0 | 20.3 | ± | 2.1 | 10.3 | 101.5 | 20.4 | ± | 2.5 | 12.3 | 102.0 |
|  | 100.0 | 112.3 | ± | 7.4 | 6.6 | 112.3 | 105.3 | ± | 5.5 | 5.2 | 105.3 | 100.8 | ± | 2.4 | 2.3 | 100.8 |
| Berberine | 2.0 | 2.3 | ± | 0.3 | 13.4 | 114.2 | 2.0 | ± | 0.1 | 3.7 | 98.8 | 1.9 | ± | 0.2 | 11.5 | 95.5 |
|  | 20.0 | 19.2 | ± | 0.3 | 1.7 | 96.2 | 19.3 | ± | 1.5 | 7.7 | 96.5 | 19.1 | ± | 2.0 | 10.5 | 95.3 |
|  | 100.0 | 103.8 | ± | 5.8 | 5.6 | 103.8 | 98.0 | ± | 4.2 | 4.3 | 98.0 | 107.9 | ± | 9.3 | 8.6 | 107.9 |
| Palmatine | 0.5 | 0.6 | ± | 0.0 | 8.4 | 110.2 | 0.5 | ± | 0.0 | 4.0 | 108.5 | 0.5 | ± | 0.1 | 13.1 | 94.9 |
|  | 5.0 | 5.1 | ± | 0.2 | 2.9 | 102.5 | 5.0 | ± | 0.1 | 2.9 | 100.1 | 4.9 | ± | 0.2 | 4.8 | 97.8 |
|  | 25.0 | 25.8 | ± | 1.6 | 6.1 | 103.2 | 23.6 | ± | 1.8 | 7.8 | 94.3 | 23.9 | ± | 0.8 | 3.1 | 95.5 |
| Coptisine | 1.0 | 1.1 | ± | 0.1 | 10.3 | 105.5 | 0.9 | ± | 0.1 | 7.2 | 89.9 | 0.9 | ± | 0.1 | 7.8 | 85.7 |
|  | 10.0 | 9.7 | ± | 0.3 | 3.1 | 96.5 | 8.7 | ± | 0.3 | 3.2 | 86.6 | 9.7 | ± | 0.4 | 4.0 | 96.6 |
|  | 50.0 | 51.1 | ± | 3.3 | 6.4 | 102.1 | 47.9 | ± | 1.5 | 3.1 | 95.8 | 47.0 | ± | 0.7 | 1.5 | 94.1 |
| Epiberberine | 2.0 | 2.1 | ± | 0.2 | 8.8 | 103.3 | 2.1 | ± | 0.3 | 14.0 | 103.0 | 1.8 | ± | 0.2 | 11.2 | 89.5 |
|  | 20.0 | 19.3 | ± | 2.1 | 11.1 | 96.3 | 18.8 | ± | 0.9 | 5.0 | 93.8 | 17.1 | ± | 1.6 | 9.5 | 85.7 |
|  | 100.0 | 109.1 | ± | 13.8 | 12.6 | 109.1 | 96.9 | ± | 13.0 | 13.4 | 96.9 | 107.3 | ± | 9.8 | 9.1 | 107.3 |
| Magnoflorine | 1.0 | 1.1 | ± | 0.0 | 3.1 | 113.3 | 0.9 | ± | 0.1 | 9.7 | 90.7 | 0.9 | ± | 0.1 | 7.1 | 89.4 |
|  | 10.0 | 9.7 | ± | 0.4 | 4.1 | 96.8 | 9.2 | ± | 1.0 | 10.8 | 91.9 | 9.1 | ± | 0.4 | 4.9 | 90.9 |
|  | 50.0 | 53.0 | ± | 2.7 | 5.1 | 106.0 | 45.8 | ± | 1.7 | 3.6 | 91.6 | 45.5 | ± | 0.6 | 1.2 | 91.1 |
| Demethylene-  berberine | 2.0 | 2.2 | ± | 0.1 | 4.4 | 109.0 | 2.0 | ± | 0.0 | 0.9 | 97.5 | 1.8 | ± | 0.1 | 5.8 | 91.7 |
|  | 20.0 | 20.8 | ± | 1.2 | 5.5 | 104.0 | 19.6 | ± | 1.6 | 8.3 | 98.2 | 20.4 | ± | 1.9 | 9.2 | 102.0 |
|  | 100.0 | 111.0 | ± | 10.6 | 9.5 | 111.0 | 90.0 | ± | 2.3 | 2.6 | 90.0 | 96.9 | ± | 2.7 | 2.8 | 96.9 |
| Berberrubine | 2.0 | 1.9 | ± | 0.1 | 3.1 | 97.3 | 1.9 | ± | 0.1 | 5.4 | 94.8 | 2.2 | ± | 0.0 | 2.2 | 111.5 |
|  | 20.0 | 19.0 | ± | 0.8 | 4.1 | 94.8 | 18.4 | ± | 1.8 | 9.7 | 92.2 | 22.6 | ± | 1.2 | 5.2 | 113.0 |
|  | 80.0 | 90.7 | ± | 8.7 | 9.6 | 113.3 | 75.8 | ± | 2.4 | 3.2 | 94.7 | 89.7 | ± | 1.8 | 2.0 | 112.2 |
| Jatrorrhizine | 1.0 | 1.1 | ± | 0.1 | 12.7 | 106.4 | 1.0 | ± | 0.1 | 14.5 | 102.1 | 0.9 | ± | 0.1 | 11.0 | 92.4 |
|  | 10.0 | 9.2 | ± | 0.8 | 8.8 | 91.8 | 9.2 | ± | 1.0 | 10.4 | 92.0 | 9.6 | ± | 0.8 | 8.5 | 95.9 |
|  | 50.0 | 54.1 | ± | 6.8 | 12.5 | 108.1 | 45.1 | ± | 3.1 | 6.8 | 90.1 | 46.4 | ± | 0.1 | 0.1 | 92.7 |
| Liquiritin | 9.0 | 8.3 | ± | 0.1 | 1.5 | 91.9 | 8.2 | ± | 0.5 | 6.0 | 90.7 | 8.7 | ± | 0.2 | 2.5 | 96.2 |
|  | 90.0 | 86.5 | ± | 2.3 | 2.7 | 96.1 | 87.5 | ± | 5.3 | 6.1 | 97.2 | 89.6 | ± | 8.9 | 10.0 | 99.5 |
|  | 320.0 | 326.3 | ± | 38.1 | 11.7 | 102.0 | 302.0 | ± | 12.1 | 4.0 | 94.4 | 311.3 | ± | 8.0 | 2.6 | 97.3 |
| Liquiritigenin | 9.0 | 9.6 | ± | 1.2 | 12.0 | 106.5 | 9.0 | ± | 0.9 | 10.2 | 100.3 | 9.0 | ± | 1.1 | 12.2 | 100.2 |
|  | 90.0 | 87.4 | ± | 5.2 | 6.0 | 97.1 | 87.0 | ± | 7.7 | 8.9 | 96.7 | 87.2 | ± | 7.7 | 8.8 | 96.9 |
|  | 320.0 | 323.3 | ± | 13.6 | 4.2 | 101.0 | 294.0 | ± | 10.1 | 3.5 | 91.9 | 313.0 | ± | 6.1 | 1.9 | 97.8 |
| Glycyrrhizic  acid | 80.0 | 91.3 | ± | 4.4 | 4.8 | 114.1 | 84.1 | ± | 2.7 | 3.2 | 105.1 | 85.7 | ± | 3.9 | 4.6 | 107.2 |
|  | 800.0 | 813.3 | ± | 10.6 | 1.3 | 101.7 | 794.3 | ± | 22.5 | 2.8 | 99.3 | 815.0 | ± | 13.1 | 1.6 | 101.9 |
|  | 3000.0 | 3120.0 | ± | 113.6 | 3.6 | 104.0 | 2910.0 | ± | 79.4 | 2.7 | 97.0 | 3330.0 | ± | 311.9 | 9.4 | 111.0 |
| Glycyrrhetinic  acid | 70.0 | 71.8 | ± | 1.7 | 2.3 | 102.6 | 67.7 | ± | 2.5 | 3.7 | 96.7 | 62.4 | ± | 1.1 | 1.7 | 89.1 |
|  | 700.0 | 710.3 | ± | 9.0 | 1.3 | 101.5 | 704.3 | ± | 6.1 | 0.9 | 100.6 | 625.7 | ± | 34.6 | 5.5 | 89.4 |
|  | 2560.0 | 2513.3 | ± | 80.8 | 3.2 | 98.2 | 2533.3 | ± | 40.4 | 1.6 | 99.0 | 2333.3 | ± | 60.3 | 2.6 | 91.1 |

Table S7. Dilution linearity of the constituents of *Gegen*-*Qinlian* decoction in blank mouse plasma (mean ± SD, n = 5).

| Constituents | Spiked  concentrations  (ng/mL) | Dilution  times | Determined  concentrations  (ng/mL) | | | RSD  (%) | RE  (%) |
| --- | --- | --- | --- | --- | --- | --- | --- |
| Puerarin | 768.0 | 10.0 | 78.5 | ± | 4.2 | 5.4 | 102.2 |
| Daidzein | 600.0 | 10.0 | 65.6 | ± | 6.4 | 9.8 | 109.4 |
| Daidzin | 480.0 | 10.0 | 49.5 | ± | 3.4 | 6.8 | 103.2 |
| Baicalin | 1530.0 | 10.0 | 157.0 | ± | 6.3 | 4.0 | 102.6 |
| Wogonoside | 1530.0 | 10.0 | 164.2 | ± | 11.4 | 7.0 | 107.3 |
| Baicalein | 1200.0 | 10.0 | 130.8 | ± | 13.2 | 10.1 | 109.0 |
| Wogonin | 300.0 | 10.0 | 34.2 | ± | 3.2 | 9.5 | 114.0 |
| Berberine | 300.0 | 10.0 | 33.4 | ± | 2.8 | 8.4 | 111.4 |
| Palmatine | 75.0 | 10.0 | 7.8 | ± | 0.4 | 4.7 | 103.7 |
| Coptisine | 150.0 | 10.0 | 16.8 | ± | 1.0 | 5.8 | 111.9 |
| Epiberberine | 300.0 | 10.0 | 32.4 | ± | 3.0 | 9.4 | 107.9 |
| Magnoflorine | 150.0 | 10.0 | 17.1 | ± | 0.8 | 4.7 | 113.9 |
| Demethyleneberberine | 300.0 | 10.0 | 33.2 | ± | 2.7 | 8.1 | 110.7 |
| Berberrubine | 240.0 | 10.0 | 27.5 | ± | 1.1 | 3.9 | 114.6 |
| Jatrorrhizine | 150.0 | 10.0 | 16.5 | ± | 1.1 | 6.5 | 109.9 |
| Liquiritin | 960.0 | 10.0 | 108.6 | ± | 11.4 | 10.5 | 113.1 |
| Liquiritigenin | 960.0 | 10.0 | 98.2 | ± | 11.5 | 11.7 | 102.2 |
| Glycyrrhizic acid | 9000.0 | 10.0 | 946.0 | ± | 36.3 | 3.8 | 105.1 |
| Glycyrrhetinic acid | 7680.0 | 10.0 | 800.8 | ± | 33.9 | 4.2 | 104.3 |

Table S8. Standard curves, linear ranges and lower limits of quantification (LLOQ) of the constituents of *Gegen-Qinlian* decoction in the blank liver homogenate of mice (n = 6).

| Constituents | Regression  equation | r | Linearity  range  (ng/mL) | LLOQ  (ng/mL) |
| --- | --- | --- | --- | --- |
| Puerarin | y=0.00796x+0.0845 | 0.9958 | 2.5-320.0 | 2.5 |
| Daidzein | y=0.0213x+0.642 | 0.9967 | 2.0-256.0 | 2.0 |
| Daidzin | y=0.0226x+0.00804 | 0.9956 | 1.6-200.0 | 1.6 |
| Baicalin | y=0.0102x+0.0465 | 0.9965 | 5.0-640.0 | 5.0 |
| Wogonoside | y=0.00524x+0.00165 | 0.9962 | 5.0-640.0 | 5.0 |
| Baicalein | y=0.0132x+0.0045 | 0.9958 | 5.0-640.0 | 5.0 |
| Wogonin | y=0.133x+0.0415 | 0.9950 | 1.0-128.0 | 1.0 |
| Berberine | y=0.126x+0.966 | 0.9955 | 1.0-128.0 | 1.0 |
| Palmatine | y=0.589x+0.95 | 0.9960 | 0.3-32.0 | 0.3 |
| Coptisine | y=0.059x+0.0829 | 0.9975 | 0.5-64.0 | 0.5 |
| Epiberberine | y=0.0366x+0.359 | 0.9967 | 1.0-128.0 | 1.0 |
| Magnoflorine | y=0.323x+0.11 | 0.9952 | 0.5-64.0 | 0.5 |
| Demethyleneberberine | y=0.0613x+0.0271 | 0.9954 | 1.0-128.0 | 1.0 |
| Berberrubine | y=0.161x-0.0135 | 0.9968 | 0.8-100.0 | 0.8 |
| Jatrorrhizine | y=0.106x+0.146 | 0.9953 | 0.5-64.0 | 0.5 |
| Liquiritin | y=0.0661x+0.121 | 0.9956 | 3.1-400.0 | 3.1 |
| Liquiritigenin | y=0.123x+1.02 | 0.9969 | 3.1-400.0 | 3.1 |
| Glycyrrhizic acid | y=0.00185x+0.0539 | 0.9956 | 25.0-3200.0 | 25.0 |
| Glycyrrhetinic acid | y=0.00399x+0.0356 | 0.9957 | 25.0-3200.0 | 25.0 |

Table S9. Intraday precision and accuracy of the constituents of *Gegen-Qinlian* decoction in quality control liver samples (mean ± SD, n = 6).

| Cconstituents | Nominal  concentrations  (ng/mL) | Determined  concentrations  (ng/mL) | | | RSD  (%) | RE  (%) |
| --- | --- | --- | --- | --- | --- | --- |
| Puerarin | 2.5 | 2.4 | ± | 0.3 | 10.4 | 97.0 |
|  | 7.0 | 7.0 | ± | 0.5 | 7.2 | 99.7 |
|  | 70.0 | 65.3 | ± | 6.6 | 10.2 | 93.3 |
|  | 256.0 | 241.2 | ± | 8.4 | 3.5 | 94.2 |
| Daidzein | 2.0 | 1.9 | ± | 0.4 | 18.8 | 94.8 |
|  | 5.0 | 5.1 | ± | 0.6 | 10.9 | 103.0 |
|  | 50.0 | 51.9 | ± | 3.1 | 6.0 | 103.8 |
|  | 200.0 | 214.0 | ± | 14.3 | 6.7 | 107.0 |
| Daidzin | 1.6 | 1.6 | ± | 0.1 | 8.2 | 101.4 |
|  | 4.0 | 3.5 | ± | 0.5 | 13.8 | 86.4 |
|  | 40.0 | 35.1 | ± | 2.6 | 7.5 | 87.8 |
|  | 160.0 | 147.7 | ± | 9.8 | 6.6 | 92.3 |
| Baicalin | 5.0 | 5.1 | ± | 0.8 | 15.5 | 102.9 |
|  | 15.0 | 15.4 | ± | 1.5 | 9.5 | 102.9 |
|  | 150.0 | 133.0 | ± | 6.4 | 4.8 | 88.7 |
|  | 510.0 | 489.3 | ± | 31.1 | 6.4 | 95.9 |
| Wogonoside | 5.0 | 5.5 | ± | 0.5 | 8.5 | 110.2 |
|  | 15.0 | 13.7 | ± | 0.8 | 6.1 | 91.0 |
|  | 150.0 | 134.2 | ± | 7.4 | 5.5 | 89.4 |
|  | 510.0 | 485.8 | ± | 35.4 | 7.3 | 95.3 |
| Baicalein | 5.0 | 5.0 | ± | 0.3 | 5.4 | 99.6 |
|  | 15.0 | 15.0 | ± | 0.5 | 3.3 | 100.0 |
|  | 150.0 | 160.0 | ± | 8.1 | 5.1 | 106.7 |
|  | 510.0 | 526.5 | ± | 12.0 | 2.3 | 103.2 |
| Wogonin | 1.0 | 1.1 | ± | 0.0 | 3.5 | 109.3 |
|  | 2.0 | 2.2 | ± | 0.3 | 11.9 | 108.2 |
|  | 20.0 | 21.7 | ± | 1.7 | 7.8 | 108.3 |
|  | 100.0 | 110.7 | ± | 8.2 | 7.4 | 110.7 |
| Berberine | 1.0 | 1.1 | ± | 0.2 | 17.8 | 106.6 |
|  | 2.0 | 2.1 | ± | 0.3 | 12.2 | 104.5 |
|  | 20.0 | 20.4 | ± | 1.7 | 8.3 | 101.9 |
|  | 100.0 | 96.0 | ± | 6.4 | 6.6 | 96.0 |
| Palmatine | 0.3 | 0.3 | ± | 0.0 | 11.6 | 102.9 |
|  | 0.5 | 0.5 | ± | 0.0 | 9.7 | 93.3 |
|  | 5.0 | 4.9 | ± | 0.5 | 10.3 | 98.1 |
|  | 25.0 | 24.6 | ± | 1.3 | 5.1 | 98.4 |
| Coptisine | 0.5 | 0.6 | ± | 0.1 | 12.3 | 110.6 |
|  | 1.0 | 1.1 | ± | 0.2 | 13.9 | 112.1 |
|  | 10.0 | 10.7 | ± | 0.9 | 8.2 | 107.0 |
|  | 50.0 | 51.2 | ± | 1.6 | 3.1 | 102.5 |
| Epiberberine | 1.0 | 1.1 | ± | 0.1 | 11.5 | 107.5 |
|  | 2.0 | 2.0 | ± | 0.1 | 5.9 | 97.6 |
|  | 20.0 | 20.4 | ± | 1.4 | 6.9 | 102.0 |
|  | 100.0 | 94.7 | ± | 6.1 | 6.4 | 94.7 |
| Magnoflorine | 0.5 | 0.5 | ± | 0.0 | 7.1 | 102.4 |
|  | 1.0 | 1.1 | ± | 0.2 | 14.9 | 107.7 |
|  | 10.0 | 10.2 | ± | 0.8 | 7.7 | 101.7 |
|  | 50.0 | 48.1 | ± | 1.6 | 3.3 | 96.3 |
| Demethyleneberberine | 1.0 | 1.1 | ± | 0.1 | 6.8 | 111.0 |
|  | 2.0 | 2.1 | ± | 0.2 | 10.8 | 103.9 |
|  | 20.0 | 19.2 | ± | 0.5 | 2.7 | 96.2 |
|  | 100.0 | 91.6 | ± | 3.7 | 4.1 | 91.6 |
| Berberrubine | 0.8 | 0.8 | ± | 0.1 | 7.1 | 102.4 |
|  | 2.0 | 1.9 | ± | 0.3 | 14.5 | 94.5 |
|  | 20.0 | 20.5 | ± | 2.1 | 10.4 | 102.3 |
|  | 80.0 | 85.6 | ± | 5.3 | 6.1 | 106.9 |
| Jatrorrhizine | 0.5 | 0.5 | ± | 0.0 | 3.3 | 104.4 |
|  | 1.0 | 0.9 | ± | 0.1 | 8.2 | 87.5 |
|  | 10.0 | 9.7 | ± | 1.2 | 12.0 | 97.0 |
|  | 50.0 | 53.2 | ± | 2.5 | 4.8 | 106.4 |
| Liquiritin | 3.1 | 3.2 | ± | 0.2 | 4.7 | 102.6 |
|  | 9.0 | 8.5 | ± | 0.4 | 4.9 | 94.8 |
|  | 90.0 | 87.4 | ± | 4.2 | 4.8 | 97.1 |
|  | 320.0 | 332.5 | ± | 21.9 | 6.6 | 103.9 |
| Liquiritigenin | 3.1 | 3.0 | ± | 0.2 | 5.3 | 96.2 |
|  | 9.0 | 9.5 | ± | 1.2 | 12.6 | 105.4 |
|  | 90.0 | 99.2 | ± | 5.2 | 5.2 | 110.2 |
|  | 320.0 | 331.3 | ± | 26.7 | 8.0 | 103.5 |
| Glycyrrhizic acid | 25.0 | 25.6 | ± | 2.4 | 9.5 | 102.3 |
|  | 70.0 | 71.5 | ± | 5.6 | 7.9 | 102.1 |
|  | 700.0 | 717.5 | ± | 51.5 | 7.2 | 102.5 |
|  | 2560.0 | 2488.3 | ± | 173.5 | 7.0 | 97.2 |
| Glycyrrhetinic acid | 25.0 | 24.9 | ± | 0.8 | 3.1 | 99.5 |
|  | 70.0 | 69.5 | ± | 5.6 | 8.1 | 99.3 |
|  | 700.0 | 698.3 | ± | 26.9 | 3.8 | 99.8 |
|  | 2560.0 | 2573.3 | ± | 124.2 | 4.8 | 100.5 |

Table S10. Carry-over of the constituents of *Gegen*-*Qinlian* decoction and internal standards (ISs) in quality control liver samples.

| Constituents | Peak area (counts) | | Carry-over (%) |
| --- | --- | --- | --- |
| Puerarin | LLOQ | 3.93E+04 | 5.1 |
|  | DB | 2.02E+03 |  |
| Daidzein | LLOQ | 2.58E+05 | 6.1 |
|  | DB | 1.58E+04 |  |
| Daidzin | LLOQ | 1.82E+04 | 3.9 |
|  | DB | 7.15E+02 |  |
| Baicalin | LLOQ | 4.20E+04 | 8.1 |
|  | DB | 3.40E+03 |  |
| Wogonoside | LLOQ | 1.26E+04 | 9.0 |
|  | DB | 1.14E+03 |  |
| Baicalein | LLOQ | 2.76E+04 | 16.4 |
|  | DB | 4.54E+03 |  |
| Wogonin | LLOQ | 7.15E+04 | 7.9 |
|  | DB | 5.64E+03 |  |
| Berberine | LLOQ | 4.35E+05 | 5.5 |
|  | DB | 2.38E+04 |  |
| Palmatine | LLOQ | 4.24E+05 | 10.3 |
|  | DB | 4.38E+04 |  |
| Coptisine | LLOQ | 4.65E+04 | 16.9 |
|  | DB | 7.84E+03 |  |
| Epiberberine | LLOQ | 1.54E+05 | 6.1 |
|  | DB | 9.47E+03 |  |
| Magnoflorine | LLOQ | 1.01E+05 | 5.9 |
|  | DB | 5.98E+03 |  |
| Demethyleneberberine | LLOQ | 3.58E+04 | 3.8 |
|  | DB | 1.37E+03 |  |
| Berberrubine | LLOQ | 4.16E+04 | 16.2 |
|  | DB | 6.72E+03 |  |
| Jatrorrhizine | LLOQ | 7.63E+04 | 8.9 |
|  | DB | 6.76E+03 |  |
| Liquiritin | LLOQ | 1.17E+04 | 4.7 |
|  | DB | 5.47E+02 |  |
| Liquiritigenin | LLOQ | 4.63E+04 | 3.3 |
|  | DB | 1.54E+03 |  |
| Glycyrrhizic acid | LLOQ | 3.42E+03 | 1.7 |
|  | DB | 5.74E+01 |  |
| Glycyrrhetinic acid | LLOQ | 4.60E+03 | 1.2 |
|  | DB | 5.32E+01 |  |
| Naringin (IS1) | LLOQ | 3.81E+05 | 0.2 |
|  | DB | 5.84E+02 |  |
| Mycophenolic acid (IS2) | LLOQ | 3.40E+04 | 0.5 |
|  | DB | 1.56E+02 |  |

LLOQ，liver matrix with lower limit of quantification；DB，double blank liver matrix

Table S11. Recovery and matrix effect of the constituents of *Gegen*-*Qinlian* decoction and internal standards (ISs) in quality control liver samples (mean ± SD, n = 6).

| Constituents | Nominal  concentrations  (ng/mL) | Recovery (%) | | | | Matrix effect (%) | | | |
| --- | --- | --- | --- | --- | --- | --- | --- | --- | --- |
|  |  | Mean | ± | SD | RSD (%) | Mean | ± | SD | RSD (%) |
| Puerarin | 7.0 | 103.8 | ± | 6.7 | 6.4 | 97.6 | ± | 7.1 | 7.3 |
|  | 70.0 | 114.1 | ± | 10.9 | 9.6 | / | | | |
|  | 256.0 | 113.6 | ± | 8.9 | 7.9 | 100.4 | ± | 4.5 | 4.5 |
| Daidzein | 5.0 | 98.1 | ± | 4.6 | 4.7 | 91.7 | ± | 4.1 | 4.5 |
|  | 50.0 | 99.4 | ± | 9.1 | 9.2 | / | | | |
|  | 200.0 | 96.6 | ± | 8.4 | 8.7 | 104.0 | ± | 4.3 | 4.1 |
| Daidzin | 4.0 | 94.9 | ± | 13.0 | 13.6 | 110.5 | ± | 11.0 | 10.0 |
|  | 40.0 | 105.3 | ± | 9.8 | 9.3 | / | | | |
|  | 160.0 | 112.9 | ± | 11.2 | 9.9 | 100.6 | ± | 7.0 | 7.0 |
| Baicalin | 15.0 | 92.5 | ± | 10.8 | 11.7 | 97.6 | ± | 10.2 | 10.5 |
|  | 150.0 | 90.4 | ± | 5.0 | 5.5 | / | | | |
|  | 510.0 | 89.4 | ± | 7.4 | 8.3 | 89.0 | ± | 4.3 | 4.9 |
| Wogonoside | 15.0 | 98.0 | ± | 8.9 | 9.1 | 90.8 | ± | 10.4 | 11.5 |
|  | 150.0 | 87.6 | ± | 8.3 | 9.5 | / | | | |
|  | 510.0 | 87.5 | ± | 5.5 | 6.2 | 94.1 | ± | 8.2 | 8.7 |
| Baicalein | 15.0 | 101.1 | ± | 4.7 | 4.7 | 104.2 | ± | 4.9 | 4.7 |
|  | 150.0 | 98.0 | ± | 12.5 | 12.8 | / | | | |
|  | 510.0 | 92.1 | ± | 5.4 | 5.8 | 107.0 | ± | 3.8 | 3.6 |
| Wogonin | 2.0 | 114.0 | ± | 9.1 | 8.0 | 112.6 | ± | 11.0 | 9.8 |
|  | 20.0 | 112.8 | ± | 11.4 | 10.1 | / | | | |
|  | 100.0 | 109.9 | ± | 13.2 | 12.0 | 101.9 | ± | 4.1 | 4.0 |
| Berberine | 2.0 | 107.4 | ± | 5.1 | 4.8 | 90.1 | ± | 5.7 | 6.3 |
|  | 20.0 | 101.8 | ± | 11.7 | 11.4 | / | | | |
|  | 100.0 | 95.0 | ± | 7.4 | 7.8 | 97.6 | ± | 4.7 | 4.8 |
| Palmatine | 0.5 | 96.4 | ± | 10.8 | 11.2 | 103.4 | ± | 4.0 | 3.8 |
|  | 5.0 | 93.2 | ± | 12.7 | 13.6 | / | | | |
|  | 25.0 | 107.3 | ± | 10.3 | 9.6 | 100.4 | ± | 3.7 | 3.7 |
| Coptisine | 1.0 | 95.1 | ± | 6.9 | 7.3 | 102.8 | ± | 8.1 | 7.9 |
|  | 10.0 | 101.8 | ± | 10.3 | 10.1 | / | | | |
|  | 50.0 | 98.5 | ± | 7.2 | 7.3 | 100.4 | ± | 2.2 | 2.2 |
| Epiberberine | 2.0 | 98.0 | ± | 14.1 | 14.3 | 98.7 | ± | 8.3 | 8.4 |
|  | 20.0 | 108.2 | ± | 8.5 | 7.9 | / | | | |
|  | 100.0 | 106.5 | ± | 3.8 | 3.6 | 105.0 | ± | 3.9 | 3.8 |
| Magnoflorine | 1.0 | 97.6 | ± | 10.9 | 11.2 | 107.1 | ± | 4.4 | 4.1 |
|  | 10.0 | 100.4 | ± | 13.4 | 13.3 | / | | | |
|  | 50.0 | 94.3 | ± | 6.7 | 7.1 | 100.5 | ± | 4.3 | 4.2 |
| Demethyleneberberine | 2.0 | 101.7 | ± | 6.9 | 6.8 | 100.4 | ± | 9.2 | 9.2 |
|  | 20.0 | 94.5 | ± | 8.3 | 8.8 | / | | | |
|  | 100.0 | 105.1 | ± | 5.6 | 5.4 | 96.6 | ± | 2.4 | 2.5 |
| Berberrubine | 2.0 | 103.4 | ± | 13.5 | 13.0 | 85.9 | ± | 9.3 | 10.8 |
|  | 20.0 | 92.5 | ± | 5.1 | 5.5 | / | | | |
|  | 80.0 | 97.1 | ± | 7.6 | 7.8 | 96.0 | ± | 4.1 | 4.3 |
| Jatrorrhizine | 1.0 | 96.8 | ± | 7.8 | 8.1 | 96.1 | ± | 10.9 | 11.4 |
|  | 10.0 | 111.3 | ± | 16.6 | 14.9 | / | | | |
|  | 50.0 | 101.5 | ± | 4.7 | 4.6 | 103.6 | ± | 5.2 | 5.0 |
| Liquiritin | 9.0 | 92.9 | ± | 5.5 | 5.9 | 90.9 | ± | 9.0 | 9.9 |
|  | 90.0 | 102.8 | ± | 8.4 | 8.1 | / | | | |
|  | 320.0 | 100.0 | ± | 5.5 | 5.5 | 87.5 | ± | 4.0 | 4.6 |
| Liquiritigenin | 9.0 | 103.7 | ± | 5.3 | 5.1 | 96.7 | ± | 6.9 | 7.1 |
|  | 90.0 | 100.7 | ± | 7.3 | 7.2 | / | | | |
|  | 320.0 | 90.7 | ± | 3.8 | 4.2 | 94.3 | ± | 3.8 | 4.0 |
| Glycyrrhizic acid | 70.0 | 98.6 | ± | 7.0 | 7.1 | 109.5 | ± | 10.3 | 9.4 |
|  | 700.0 | 87.5 | ± | 8.7 | 9.9 | / | | | |
|  | 2560.0 | 91.6 | ± | 7.7 | 8.5 | 88.5 | ± | 5.9 | 6.6 |
| Glycyrrhetinic acid | 70.0 | 97.9 | ± | 8.6 | 8.8 | 107.4 | ± | 11.0 | 10.3 |
|  | 700.0 | 94.8 | ± | 5.4 | 5.7 | / | | | |
|  | 2560.0 | 99.6 | ± | 6.0 | 6.0 | 98.2 | ± | 5.9 | 6.0 |
| Naringin (IS1) | 20000.0 | 99.9 | ± | 4.0 | 4.0 | 85.5 | ± | 1.6 | 1.9 |
|  |  | 97.7 | ± | 10.3 | 10.6 | / | | | |
|  |  | 94.0 | ± | 5.5 | 5.8 | 100.4 | ± | 4.0 | 4.0 |
| Mycophenolic acid (IS2) | 150.0 | 101.2 | ± | 4.2 | 4.2 | 107.8 | ± | 6.7 | 6.2 |
|  |  | 100.4 | ± | 4.9 | 4.9 | / | | | |
|  |  | 103.4 | ± | 5.6 | 5.4 | 97.7 | ± | 3.1 | 3.1 |

Table S12. Stability of the constituents of *Gegen*-*Qinlian* decoction in quality control liver samples (mean ± SD, n = 3).

| Constituents | Nominal concentrations (ng/mL) | -80°C for 7days | | | | | 4°C for 24 h | | | | | 22°C for 2 h | | | | |
| --- | --- | --- | --- | --- | --- | --- | --- | --- | --- | --- | --- | --- | --- | --- | --- | --- |
|  |  | Determined concentrations (ng/mL) | | | RSD (%) | RE (%) | Determined concentrations (ng/mL) | | | RSD (%) | RE (%) | Determined concentrations (ng/mL) | | | RSD (%) | RE (%) |
| Puerarin | 7.0 | 6.5 | ± | 0.3 | 3.9 | 92.2 | 7.3 | ± | 0.4 | 4.8 | 104.9 | 6.6 | ± | 0.6 | 9.4 | 94.9 |
|  | 256.0 | 237.3 | ± | 19.5 | 8.2 | 92.7 | 242.0 | ± | 4.4 | 1.8 | 94.5 | 248.0 | ± | 25.2 | 10.2 | 96.9 |
| Daidzein | 5.0 | 5.3 | ± | 0.7 | 13.4 | 106.7 | 5.2 | ± | 0.3 | 5.6 | 104.5 | 5.3 | ± | 0.8 | 14.3 | 106.9 |
|  | 200.0 | 199.0 | ± | 8.9 | 4.5 | 99.5 | 185.7 | ± | 3.8 | 2.0 | 92.8 | 210.3 | ± | 19.5 | 9.3 | 105.2 |
| Daidzin | 4.0 | 3.9 | ± | 0.2 | 4.0 | 98.0 | 3.6 | ± | 0.2 | 4.5 | 91.1 | 3.9 | ± | 0.2 | 4.8 | 97.6 |
|  | 160.0 | 154.0 | ± | 9.6 | 6.3 | 96.3 | 144.3 | ± | 8.1 | 5.6 | 90.2 | 156.3 | ± | 16.7 | 10.7 | 97.7 |
| Baicalin | 15.0 | 14.5 | ± | 1.0 | 6.6 | 96.7 | 15.5 | ± | 2.1 | 13.5 | 103.3 | 14.2 | ± | 1.5 | 10.6 | 94.9 |
|  | 510.0 | 519.7 | ± | 14.2 | 2.7 | 101.9 | 471.0 | ± | 20.8 | 4.4 | 92.4 | 508.0 | ± | 20.4 | 4.0 | 99.6 |
| Wogonoside | 15.0 | 15.3 | ± | 0.5 | 3.0 | 102.0 | 13.9 | ± | 1.0 | 7.3 | 92.7 | 15.3 | ± | 1.0 | 6.8 | 101.8 |
|  | 510.0 | 502.0 | ± | 29.3 | 5.8 | 98.4 | 482.7 | ± | 36.4 | 7.5 | 94.6 | 505.7 | ± | 17.4 | 3.4 | 99.2 |
| Baicalein | 15.0 | 15.0 | ± | 0.8 | 5.2 | 100.0 | 14.9 | ± | 0.6 | 3.9 | 99.6 | 15.7 | ± | 1.0 | 6.1 | 104.7 |
|  | 510.0 | 519.3 | ± | 2.1 | 0.4 | 101.8 | 523.7 | ± | 15.9 | 3.0 | 102.7 | 509.7 | ± | 19.9 | 3.9 | 99.9 |
| Wogonin | 2.0 | 2.1 | ± | 0.2 | 10.3 | 107.0 | 2.1 | ± | 0.3 | 13.5 | 106.0 | 2.0 | ± | 0.1 | 6.9 | 102.0 |
|  | 100.0 | 91.1 | ± | 3.6 | 4.0 | 91.1 | 113.0 | ± | 5.6 | 4.9 | 113.0 | 86.9 | ± | 6.1 | 7.1 | 86.9 |
| Berberine | 2.0 | 2.1 | ± | 0.2 | 8.2 | 105.2 | 2.1 | ± | 0.3 | 12.1 | 103.7 | 2.0 | ± | 0.0 | 1.1 | 99.3 |
|  | 100.0 | 91.6 | ± | 2.1 | 2.3 | 91.6 | 96.2 | ± | 4.8 | 5.0 | 96.2 | 101.5 | ± | 6.4 | 6.3 | 101.5 |
| Palmatine | 0.5 | 0.6 | ± | 0.1 | 10.7 | 110.7 | 0.5 | ± | 0.1 | 11.7 | 93.8 | 0.6 | ± | 0.0 | 8.8 | 110.5 |
|  | 25.0 | 24.6 | ± | 0.6 | 2.3 | 98.4 | 23.9 | ± | 1.2 | 4.9 | 95.5 | 25.4 | ± | 0.7 | 2.8 | 101.7 |
| Coptisine | 1.0 | 0.9 | ± | 0.0 | 4.1 | 92.4 | 1.0 | ± | 0.1 | 12.8 | 104.4 | 1.0 | ± | 0.1 | 10.4 | 101.6 |
|  | 50.0 | 49.0 | ± | 2.4 | 4.9 | 97.9 | 51.5 | ± | 1.6 | 3.1 | 102.9 | 52.3 | ± | 1.1 | 2.2 | 104.5 |
| Epiberberine | 2.0 | 2.1 | ± | 0.2 | 10.9 | 105.0 | 1.9 | ± | 0.0 | 1.7 | 94.8 | 1.9 | ± | 0.2 | 11.3 | 95.2 |
|  | 100.0 | 93.0 | ± | 6.3 | 6.8 | 93.0 | 96.3 | ± | 6.3 | 6.5 | 96.3 | 92.8 | ± | 10.3 | 11.1 | 92.8 |
| Magnoflorine | 1.0 | 0.9 | ± | 0.1 | 6.0 | 85.7 | 1.0 | ± | 0.1 | 5.2 | 97.7 | 1.0 | ± | 0.1 | 9.6 | 98.4 |
|  | 50.0 | 48.2 | ± | 2.7 | 5.6 | 96.4 | 47.8 | ± | 1.6 | 3.3 | 95.5 | 47.5 | ± | 2.6 | 5.4 | 95.1 |
| Demethylene-  berberine | 2.0 | 1.9 | ± | 0.3 | 14.1 | 97.3 | 1.9 | ± | 0.1 | 5.8 | 94.3 | 1.9 | ± | 0.1 | 7.4 | 97.3 |
|  | 100.0 | 88.0 | ± | 6.4 | 7.3 | 88.0 | 90.6 | ± | 5.7 | 6.2 | 90.6 | 93.2 | ± | 7.7 | 8.2 | 93.2 |
| Berberrubine | 2.0 | 1.7 | ± | 0.2 | 10.6 | 86.0 | 1.7 | ± | 0.1 | 7.2 | 86.2 | 2.0 | ± | 0.2 | 11.3 | 100.3 |
|  | 80.0 | 82.2 | ± | 3.5 | 4.2 | 102.8 | 82.2 | ± | 4.9 | 6.0 | 102.7 | 82.4 | ± | 4.3 | 5.2 | 103.0 |
| Jatrorrhizine | 1.0 | 0.9 | ± | 0.1 | 15.0 | 93.8 | 0.9 | ± | 0.1 | 9.3 | 86.0 | 0.9 | ± | 0.1 | 10.6 | 94.0 |
|  | 50.0 | 48.4 | ± | 2.5 | 5.1 | 96.9 | 52.2 | ± | 3.5 | 6.7 | 104.5 | 50.4 | ± | 2.8 | 5.6 | 100.9 |
| Liquiritin | 9.0 | 8.8 | ± | 0.2 | 2.6 | 97.3 | 8.2 | ± | 0.2 | 2.7 | 91.3 | 9.3 | ± | 0.4 | 4.3 | 102.9 |
|  | 320.0 | 324.3 | ± | 10.0 | 3.1 | 101.4 | 329.7 | ± | 31.2 | 9.5 | 103.0 | 315.3 | ± | 12.6 | 4.0 | 98.5 |
| Liquiritigenin | 9.0 | 9.0 | ± | 0.3 | 3.2 | 100.1 | 9.8 | ± | 1.0 | 10.2 | 108.6 | 8.8 | ± | 0.6 | 6.6 | 97.3 |
|  | 320.0 | 332.0 | ± | 17.4 | 5.3 | 103.8 | 334.3 | ± | 30.9 | 9.2 | 104.5 | 334.7 | ± | 7.4 | 2.2 | 104.6 |
| Glycyrrhizic acid | 70.0 | 73.2 | ± | 6.6 | 9.0 | 104.5 | 70.1 | ± | 5.8 | 8.3 | 100.1 | 70.7 | ± | 6.2 | 8.8 | 101.0 |
|  | 2560.0 | 2413.3 | ± | 136.1 | 5.6 | 94.3 | 2483.3 | ± | 171.6 | 6.9 | 97.0 | 2703.3 | ± | 250.1 | 9.3 | 105.6 |
| Glycyrrhetinic acid | 70.0 | 70.2 | ± | 4.6 | 6.5 | 100.3 | 66.6 | ± | 2.7 | 4.0 | 95.1 | 70.5 | ± | 2.7 | 3.8 | 100.7 |
|  | 2560.0 | 2563.3 | ± | 86.2 | 3.4 | 100.1 | 2556.7 | ± | 122.2 | 4.8 | 99.9 | 2583.3 | ± | 155.0 | 6.0 | 100.9 |

**3.1 Supplementary figures**

**
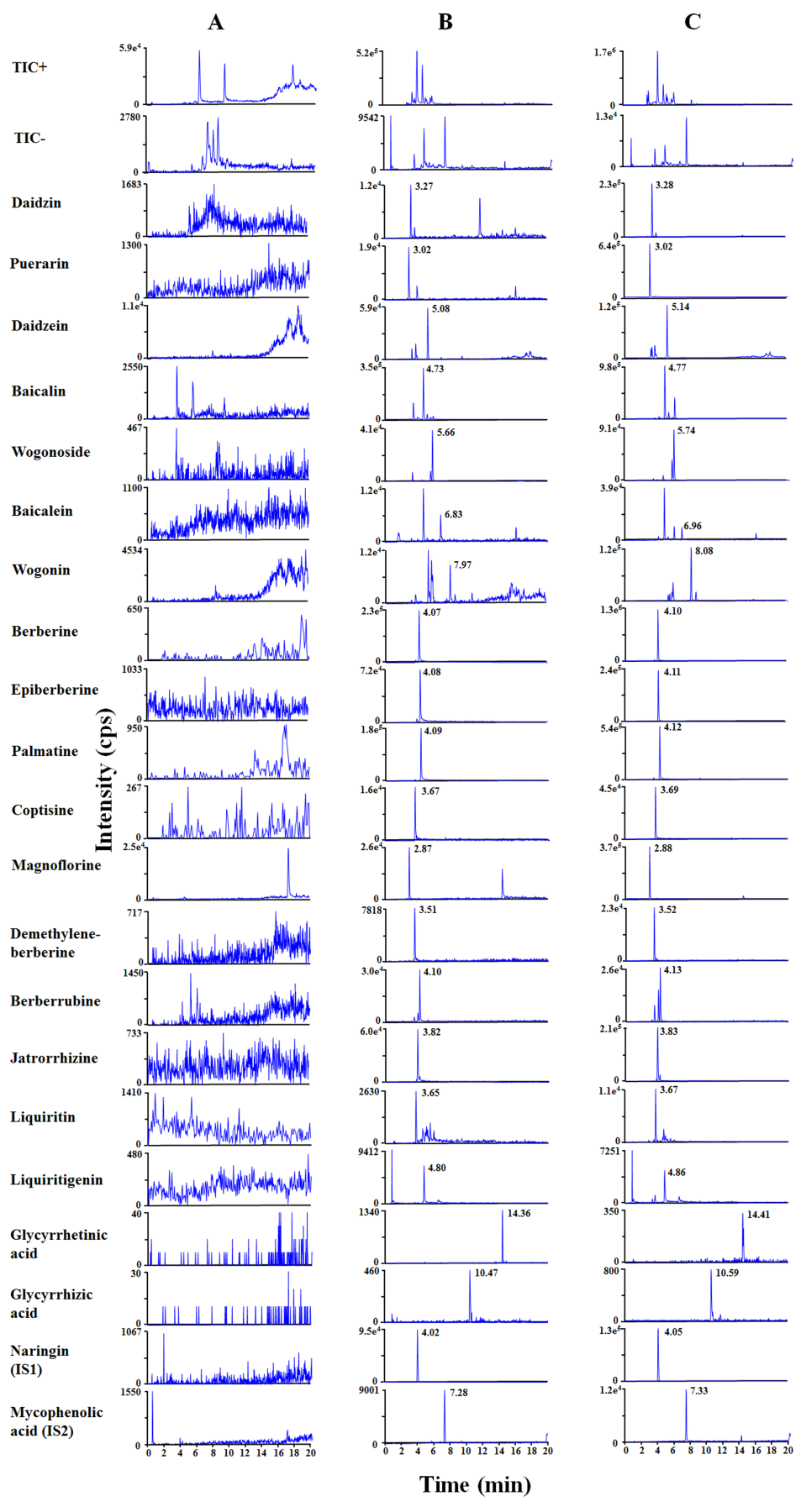
**

Figure S1. Representative total and extracted ion chromatograms of the constituents of *Gegen*-*Qinlian* decoction and internal standards (ISs) in different plasma. A, blank plasma; B, blank plasma spiked with the lower limit of quantification of the constituents; C, plasma samples obtained 1 h after oral administration of *Gegen*-*Qinlian* decoction. TIC+, total ion chromatograms in positive mode; TIC-, total ion chromatograms in negative mode.

**
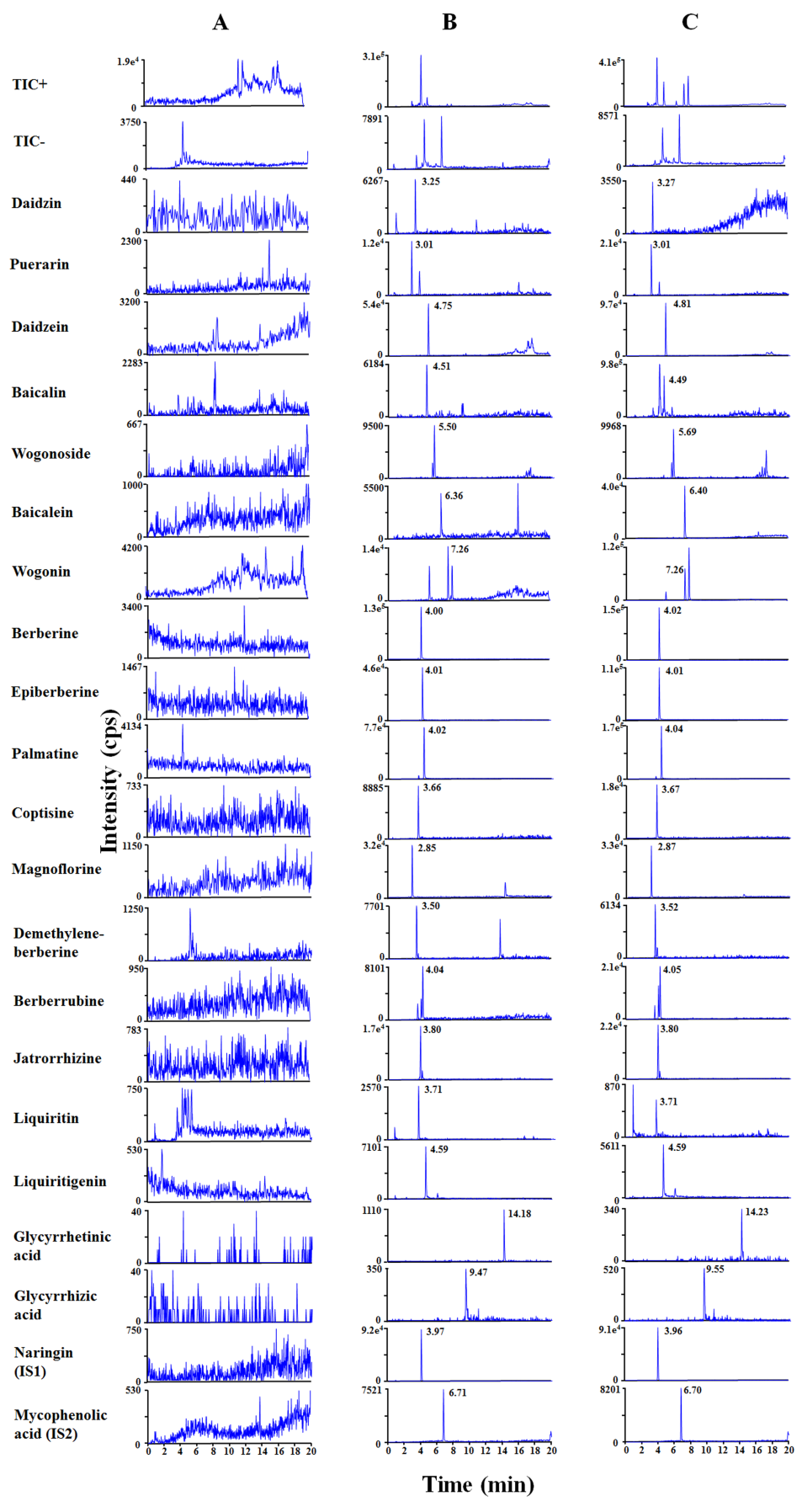
**

Figure S2. Representative total and extracted ion chromatograms of the constituents of *Gegen*-*Qinlian* decoction and internal standards (ISs) in different liver homogenate. A, blank liver homogenate; B, blank liver homogenate spiked with the lower limit of quantification of the constituents; C, liver homogenate samples obtained 1 h after oral administration of *Gegen*-*Qinlian* decoction. TIC+, total ion chromatograms in positive mode; TIC-, total ion chromatograms in negative mode.


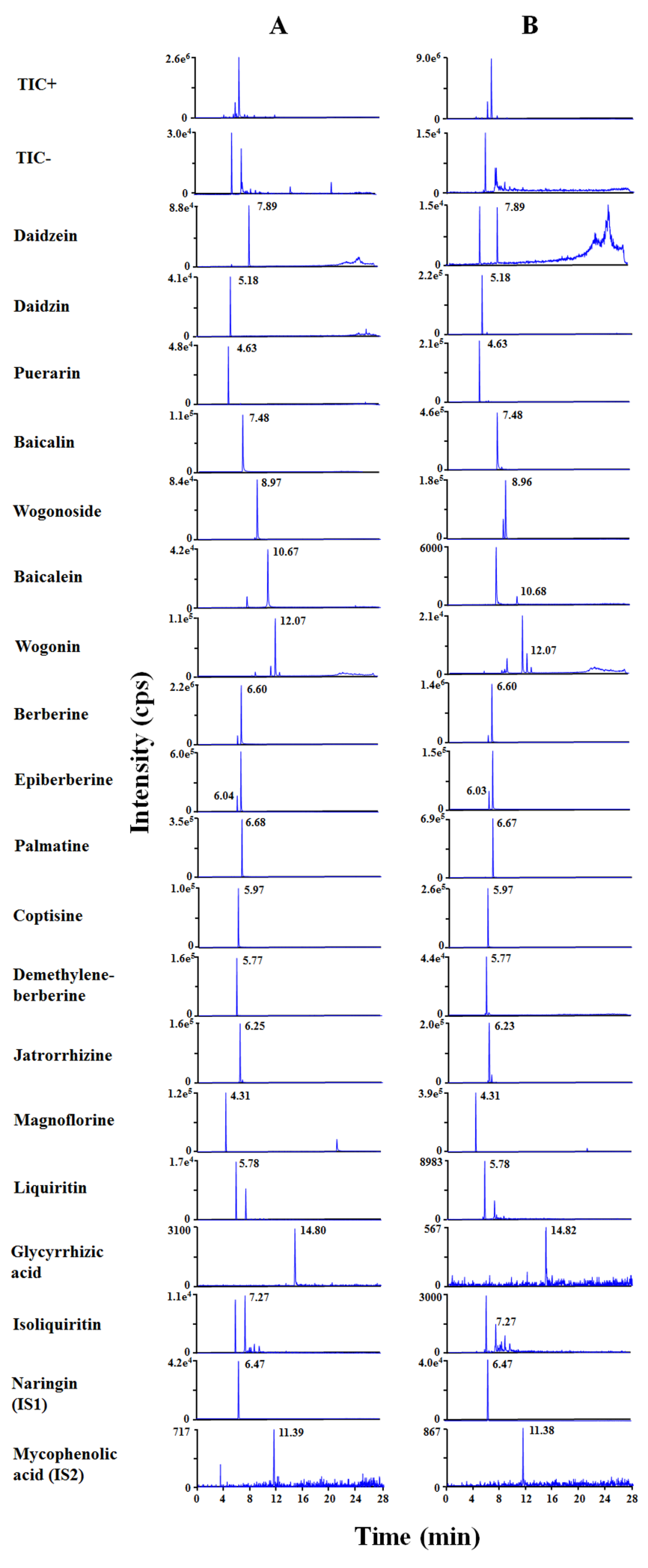


Figure S3. Representative total and extracted ion chromatograms of the constituents of *Gegen*-*Qinlian* decoction and internal standards (ISs) in water solution. A, water spiked with the lower limit of quantification of the constituents; C, water solution of the *Gegen*-*Qinlian* decoction. TIC+, total ion chromatograms in positive mode; TIC-, total ion chromatograms in negative mode.
